# Factors associated with acute kidney injury in acute respiratory distress syndrome

**DOI:** 10.1186/s13613-019-0552-5

**Published:** 2019-07-01

**Authors:** Anupol Panitchote, Omar Mehkri, Andrei Hastings, Tarik Hanane, Sevag Demirjian, Heather Torbic, Eduardo Mireles-Cabodevila, Sudhir Krishnan, Abhijit Duggal

**Affiliations:** 1 Department of Critical Care, Respiratory Institute, Cleveland Clinic, Cleveland, OH USA; 2 Division of Critical Care Medicine, Department of Medicine, Faculty of Medicine, Khon Kaen University, Khon Kaen, Thailand; 3 Department of Nephrology, Cleveland Clinic, Cleveland, OH USA; 4 Department of Pharmacology, Cleveland Clinic, Cleveland, OH USA

**Keywords:** Acute respiratory distress syndrome, Acute kidney injury, Mechanical ventilation, Gas exchange, Lung protective ventilation, Septic shock

## Abstract

**Background:**

Acute kidney injury (AKI) is the most frequent extra-pulmonary organ failure in acute respiratory distress syndrome (ARDS). The objective of this study was to assess the factors associated with the development and severity of AKI in patients with ARDS.

**Methods:**

This is a retrospective cohort study of ARDS patients without acute or chronic kidney disease prior to the onset of ARDS over a 7-year period (2010–2017). AKI and severity of AKI were defined according to the Kidney Disease Improving Global Outcomes 2012 guidelines.

**Results:**

Of the 634 ARDS patients, 357 patients met study criteria. A total of 244 (68.3%) patients developed AKI after ARDS onset: 60 (24.6%) had stage I AKI, 66 (27%) had stage II AKI, and 118 (48.4%) had stage III AKI. The median time of AKI onset for stage I AKI was 2 days (interquartile range, 1.5–5.5) while stage II and III AKI was 4 days. On multivariable analysis, factors associated with development of AKI were age [subdistribution hazard ratio (SHR) 1.01, 95% confidence interval (CI) 1.00–1.02], SOFA score (SHR 1.16, 95%CI 1.12–1.21), a history of diabetes mellitus (DM) (SHR 1.42, 95%CI 1.07–1.89), and arterial pH on day 1 of ARDS (SHR per 0.1 units decrease was 1.18, 95%CI 1.05–1.32). In severity of AKI, stage I AKI was associated with age (SHR 1.03, 95%CI 1.01–1.05) and serum bicarbonate on day 1 of ARDS (SHR 1.07, 95%CI 1.02–1.13). Stage II AKI was associated with age (SHR 1.03, 95%CI 1.01–1.05), serum bicarbonate on day 1 (SHR 1.12, 95%CI 1.06–1.18), SOFA score (SHR 1.19, 95%CI 1.10–1.30), history of heart failure (SHR 3.71, 95%CI 1.63–8.46), and peak airway pressure (SHR 1.04, 95%CI 1.00–1.07). Stage III AKI was associated with a higher BMI (SHR 1.02, 95%CI 1.00–1.03), a history of DM (SHR 1.79, 95%CI 1.18–2.72), SOFA score (SHR 1.29, 95%CI 1.22–1.36), and arterial pH on day 1 (SHR per 0.1 units decrease was 1.25, 95%CI 1.05–1.49).

**Conclusions:**

Age, a higher severity of illness, a history of diabetes, and acidosis were associated with development of AKI in ARDS patients. Severity of AKI was further associated with BMI, history of heart failure, and peak airway pressure.

**Electronic supplementary material:**

The online version of this article (10.1186/s13613-019-0552-5) contains supplementary material, which is available to authorized users.

## Background

Acute respiratory distress syndrome (ARDS) is associated with a high mortality [1]. The systemic inflammatory response associated with ARDS not only causes lung injury, but also significantly impacts the cardiovascular, renal, and neurologic function [2–4]. Acute kidney injury (AKI) is the most frequent extra-pulmonary organ dysfunction associated with ARDS and affects almost 50% of the patients. Development of AKI is an independent risk factor for mortality in patients with ARDS [5, 6]. In critically ill patients, age, presence of ARDS, shock, comorbidities such as diabetes mellitus (DM), immunosuppression, chronic liver disease, chronic heart disease, and hematologic malignancies have all been identified as risk factors to the development of AKI [7].

Several investigative studies have suggested that mechanical ventilation and ARDS may have significant adverse effects on renal function, but few studies have specifically addressed the effect of ARDS on renal function [8]. The mechanism of renal injury in ARDS is not completely understood, but experimental studies have suggested many potential mechanisms might be at play in the propagation of AKI in ARDS patients [9]. High intrathoracic pressures as a consequence of ventilator interaction with poorly compliant lungs can reduce cardiac output which results in inadequate renal perfusion; subsequent gas exchange abnormalities resulting in hypoxemia, hypercarbia, and systemic acidosis could influence renal vascular resistance altering renal perfusion pressures, resulting in AKI [10, 11]. Similarly ventilator-induced lung injury (VILI) propagates multi-organ failure due to an ongoing release of inflammatory cytokines [12], and VILI, itself, further impacts kidney function via hemodynamic compromise and neurohormonal alterations [13, 14]. Based on these mechanisms, factors that augment transmission of intrathoracic pressure to the vasculature, non-protective ventilator settings, or higher ventilator pressures have been postulated as risk factors for renal compromise [15, 16].

There are no clinical studies in ARDS that have evaluated the impact of mechanical ventilation settings on the development of AKI. Also, confirmation of risk factors identified in the development of AKI in critical illness remains to be elucidated in patients with ARDS [7]. The primary objective of this study was to identify variables associated with the development and severity of AKI in patients with ARDS.

## Methods

### Study setting and patient population

We conducted a retrospective cohort study from January 1, 2010, to May 31, 2017. The study was conducted at the Cleveland Clinic that is a quaternary academic referral center. We included all adult (> 18 years old) patients admitted to a medical intensive care unit (ICU) with a diagnosis of ARDS based on the Berlin definition [17]. We excluded patients with preexisting chronic kidney disease [defined as an estimated glomerular filtration rate (GFR) less than 60 mL/min/1.73 m^2^] [18], AKI prior to the onset of ARDS, or ICU stay < 24 h. This study was approved by the Cleveland Clinic Institutional Review Board.

### Data collection and definition

AKI and severity of AKI were defined according to the Kidney Disease Improving Global Outcomes (KDIGO) 2012 guidelines, using serum creatinine (SCr) and urine output criteria [19]. Baseline SCr values were assessed using the mean value between 7 and 365 days before hospitalization [20]. In patients where baseline renal function was not available, the baseline SCr was imputed by using the Modification of Diet in Renal Disease (MDRD) equation for a normal GFR of 75 mL/min per 1.73 m^2^ [21]. We divided patients into four groups (no AKI, stage I AKI, stage II AKI, and stage III AKI).

Collected data were extracted from electronic medical records. Day 1 was defined as the first day that patient met criteria of ARDS, irrespective of ICU admission date [1]. Demographic data included: age, sex, ethnicity, race, body mass index (BMI), comorbidities, Charlson comorbidity index, ARDS risk factors, echocardiographic findings and outside hospital transfer. Severity of illness including the sequential organ failure assessment (SOFA) score and the acute physiology, age, chronic health evaluation (APACHE) III score were recorded on day 1 of ARDS. For outside transfers, SOFA and APACHE III scores were recorded in the first 24 h of hospital admission. Mechanical ventilation parameters, arterial blood gas, serum lactate, serum bicarbonate, intake, output, and percentage of fluid overload were collected for the first three days, day 7, and day 14 of onset of ARDS. Percentage of fluid overload was calculated using the following formula [22]: Percentage of fluid overload (%) = [fluid intake (L) − total output (L)]/body weight at day 1 of ARDS (kg) × 100. Serum creatinine, urine volume, and use of renal replacement therapy (RRT) were recorded until 28 days after ARDS diagnosis or hospital discharge in order to determine the highest stage of AKI. For patients with ARDS who developed AKI, SCr, urine volume, and use of RRT were recorded until 28 days after AKI diagnosis or hospital discharge. Patients who had did not have AKI during hospital admission and were discharged with alive before day 28 were expected that they did not have AKI. Exposure to common nephrotoxic agents including but not limited to (e.g., contrast media, antimicrobial agents), septic shock, and vasopressor use were recorded daily until day 28 of ARDS. Septic shock was defined according to the Sepsis-3 consensus definition [23]. The primary outcome of interest was the development of AKI. The secondary outcomes studied included severity of AKI up to 28 days after ARDS diagnosis and survival at day 28 and 90 stratified by AKI severity. Study data were collected and managed using REDCap [24].

### Statistical analysis

Continuous variables were presented as mean (standard deviation) or median [interquartile range (IQR)] as appropriate. Categorical variables were described as counts and percentages (%). Two-sample *t* test, Wilcoxon rank sum, ANOVA, or Kruskal–Wallis test was used to compare continuous variables as appropriate. Chi-square test was used for categorical variables. Prior to the analyses, the missing data were handled using multiple imputations [25]. The imputation process is shown in Additional file 1: Appendix 1.

Factors associated with development of AKI and severity of AKI were analyzed by competing risk regression with Fine–Gray model in that death at day 28 was treated as competing risk. The effects of covariates on development of AKI were shown as subdistribution hazard ratios (SHRs). In addition, time of development of ARDS was considered for the clustering using a gamma random effects frailty model. Multivariable Cox proportional hazards regression was performed to compare the four groups of AKI for the 28-day and 90-day survival. Examination of Schoenfeld residuals indicated that the proportional hazards assumption was not violated. The adjusted survival curve was generated for the estimation of 28-day survival by AKI severity. To build a multivariable regression model, univariable regression was first performed. The variables significant at *p* < 0.1 on univariable analysis were identified as potential predictor variables and entered into a multivariable regression model. Variable selection techniques included two steps: The first step involved performing backward and forward stepwise model selection based on the Akaike information criterion (AIC) separately on each imputed dataset, followed by the construction of a new supermodel that contained all variables that were present in at least half of the initial models. Second, a special procedure for backward elimination was applied to all variables present in the supermodel. The pooled likelihood ratio *p* value was calculated. If the largest *p* value is larger than 0.05, the corresponding variable was removed, and the procedure was repeated on the smaller model. The procedure stops if all *p* ≤ 0.05.

We performed a subgroup analysis on patients who did not have septic shock, as this group of patients did not have significant confounding in terms of AKI development as a consequence of sepsis-induced multi-organ failure. We also performed sensitivity analysis using non-imputed data. All the statistical analyses were performed by using R software version 3.6.0. The level of statistical significance was set at *p* < 0.05 (two tailed).

## Results

**Fig. 1:**
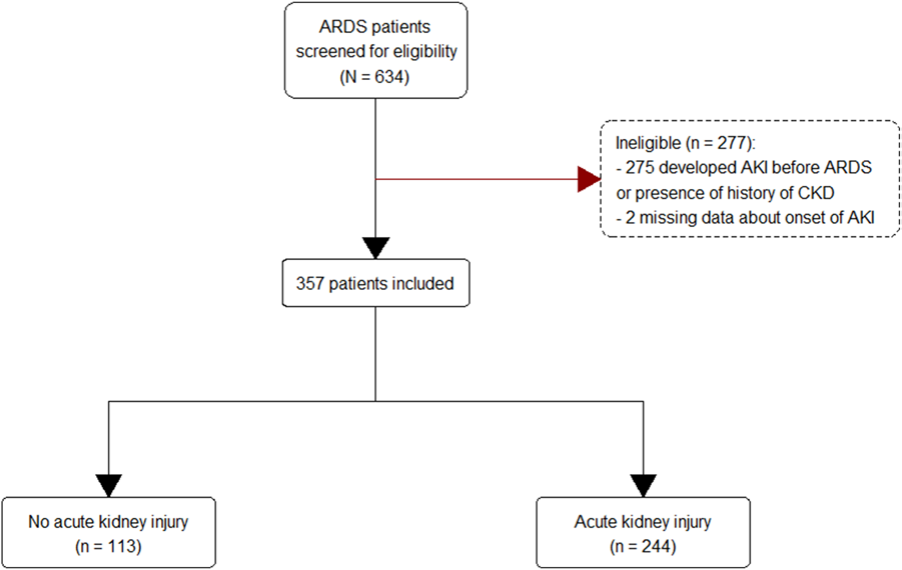
Consort flow chart

Of the 634 patients with a diagnosis of ARDS, 357 patients met inclusion criteria for our study. The consort flowchart is shown in Fig. 1. Mean patient age was 53 ± 15.8 years. Pneumonia was the primary cause of ARDS in 83% of the patients, and 50% of the patients developed septic shock at some point during their ICU stay. Two hundred and forty-four patients (68.3%) developed AKI: 60 patients (24.6%) developed stage I AKI, 66 patients (27%) developed stage II AKI, and 118 patients (48.4%) developed stage III AKI. Median time of AKI onset based on the KDIGO classification was 3 days after the diagnosis of ARDS (IQR, 2–7). Stage I AKI developed earlier 2 (IQR, 1.5–5.5), when compared to both stage II AKI (4 (2–7.8)) and stage III AKI (4 (3–7)).

### Factors associated with development of AKI

Patients with a diagnosis of AKI had a significantly higher age, severity of illness, and comorbidity index. AKI patients also had a greater prevalence of liver disease, extra-pulmonary causes of ARDS, and presence of septic shock (Table 1) (Additional file 1: Table S3). There was no association between types or number of nephrotoxic agents and development of AKI among ARDS patients (Table 1). In addition, patients with AKI received higher minute ventilation, had a higher prevalence of metabolic acidosis on day 1 of ARDS, and had a higher amount of fluid overload, while having a lower partial pressure of carbon dioxide (PaCO_2_). Patients who received lung protective strategies (tidal volume ≤ 8 mL per kg PBW and inspiratory plateau pressure ≤ 30 cm H_2_O) did not have an association with the development of AKI (Table 2) (Additional file 1: Table S3).

**Table 1:** Baseline characteristics by acute kidney injury

Characteristics	No AKI (113)	AKI (244)	*p*	Missing (%)
Age, mean (SD), years	50.2 (15.8)	54.3 (15.7)	0.02^a^	0 (0)
Male sex, *n* (%)	54 (47.8)	137 (56.1)	0.14	0 (0)
BMI, median (IQR), kg/m^2^	29.7 (24.6–35.5)	30.3 (25–38.2)	0.36	0 (0)
Race, *n* (%)				
White	88 (77.9)	170 (69.7)	0.11	0 (0)
Black or African-American	20 (17.7)	58 (23.8)	0.20	0 (0)
SOFA, mean (SD), points	9 (2.6)	11.9 (3.6)	< 0.001^a^	9 (2.5)
Non-renal SOFA, mean (SD), points	8.9 (2.6)	10.7 (3)	< 0.001^a^	9 (2.5)
APACHE III, mean (SD), points	95 (26)	115 (31)	< 0.001^a^	15 (4.2)
Charlson comorbidities index, median (IQR), points	2 (0–4)	3 (1–5)	0.002^a^	0 (0)
Comorbidities, *n* (%)				
Chronic lung diseases	44 (38.9)	74 (30.3)	0.11	0 (0)
Diabetes	23 (20.4)	72 (29.5)	0.07	0 (0)
Active malignancies	18 (15.9)	59 (24.2)	0.08	0 (0)
Liver disease	5 (4.4)	28 (11.5)	0.03^a^	0 (0)
Heart failure	5 (4.4)	22 (9)	0.13	0 (0)
Recent surgery within 3 months	4 (3.5)	10 (4.1)	1.00	0 (0)
Cause of ARDS, *n* (%)				
Pneumonia	91 (80.5)	204 (83.6)	0.48	0 (0)
Aspiration	22 (19.5)	43 (17.6)	0.67	0 (0)
Non-pulmonary sepsis	3 (2.7)	21 (8.6)	0.04^a^	0 (0)
Pancreatitis	6 (5.3)	8 (3.3)	0.39	0 (0)
Echocardiographic findings				
EF, median (IQR), %	60 (55–65)	60 (55–65)	0.21	46 (12.9)
RVSP, median (IQR), mm Hg	38 (31–48)	40 (32–50)	0.33	123 (34.5)
Septic shock	37 (32.7)	142 (58.2)	< 0.001^a^	0 (0)
Nephrotoxic agents, *n* (%)				
Antimicrobial agents	103 (91.2)	195 (84.4)	0.08	13 (3.6)
Contrast agents	34 (30.1)	64 (26.3)	0.46	1 (0.3)
ACEI	15 (13.3)	27 (11.1)	0.55	0 (0)
Calcineurin inhibitors	5 (4.4)	10 (4.1)	1.00	0 (0)
NSAIDs	4 (3.5)	4 (1.6)	0.27	0 (0)
Number of nephrotoxic agents, median (IQR), count	2 (1–3)	2 (1–3)	0.75	13 (3.6)
Rescue therapies (%)				
Continuous NMBA	37 (32.7)	99 (40.6)	0.16	0 (0)
Inhaled vasodilators	21 (18.6)	64 (26.2)	0.11	0 (0)
Prone positioning	13 (11.5)	34 (13.9)	0.53	0 (0)
ECMO	5 (4.4)	7 (2.9)	0.53	0 (0)
Recruitment maneuvers	4 (3.5)	20 (8.2)	0.10	0 (0)
HFOV	2 (1.8)	11 (4.5)	0.24	0 (0)
Known baseline SCr, *n* (%)	57 (50.4)	145 (59.4)	0.11	0 (0)
Baseline SCr, mean (SD), mg/dL	0.79 (0.18)	0.82 (0.2)	0.44	156 (43.7)
eGFR, median (IQR), mL/min per 1.73 m^2^	98.2 (80.7–111.8)	95.7 (80.7–111.1)	0.83	156 (43.7)
Time from ARDS diagnosis to hospital admission (IQR), days	0 (− 3 to 1)	0 (− 2 to 2)	0.14	0 (0)

*ACEI* angiotensin-converting enzyme inhibitors, *AKI* acute kidney injury, *APACHE* acute physiology, age, chronic health evaluation, *ARDS* acute respiratory distress syndrome, *BMI* body mass index, *ECMO* extracorporeal membrane oxygenation, *EF* ejection fraction, *eGFR* estimated glomerular filtration rate, *HFOV* high-frequency oscillatory ventilation, *IQR* interquartile range, *NMBA* neuromuscular blocking agents, *NSAIDs* nonsteroidal anti-inflammatory drugs, *RVSP* right ventricular systolic pressure, *SCr* serum creatinine, *SD* standard deviation, *SOFA* sequential organ failure assessment

^a^*p* < 0.05

**Table 2:** Ventilator settings averaged on day 1–3, arterial blood gas, and fluid overload in patients with and without acute kidney injury

Ventilator settings	No AKI (113)	AKI (244)	*p*	Missing (%)
VT, median (IQR), mL	451 (401–502)	487 (422–551)	0.01^a^	35 (9.8)
VT, median (IQR), (mL/kg PBW)	7.4 (6.7–8.6)	7.5 (6.8–8.5)	0.89	35 (9.8)
PEEP, median (IQR), cm H_2_O	10.7 (8–13.3)	10 (8–14)	0.57	32 (9.0)
FiO_2_, median (IQR)	0.67 (0.54–0.8)	0.7 (0.57–0.87)	0.23	29 (8.1)
Pplat, median (IQR), cm H_2_O	27 (23–31)	27.2 (22.9–33.1)	0.38	158 (44.3)
Pplat > 30 cm H_2_O (%)	18 (30.5)	51 (36.4)	0.42	158 (44.3)
DP, median (IQR), cm H_2_O	15 (11.3–18.5)	14.8 (12–19)	0.82	158 (44.3)
Mean airway pressure, median (IQR), cm H_2_O	17.3 (14.3–20.7)	18 (14.7–21.5)	0.16	66 (18.5)
Ppeak, mean (SD), cm H_2_O	29.6 (6.5)	31.2 (7.3)	0.08	62 (17.4)
Minute ventilation, median (IQR), L/min	10.7 (8.5–11.9)	11.4 (9.7–13.1)	0.01^a^	63 (17.6)
Lung protective ventilation, *n* (%)^b^	26 (35.1)	64 (36.2)	0.88	106 (29.7)
Arterial blood gases				
Arterial pH, median (IQR)	7.39 (7.34–7.43)	7.35 (7.3–7.41)	< 0.001^a^	28 (7.8)
Arterial pH on day 1	7.38 (7.32–7.42)	7.34 (7.27–7.4)	0.005^a^	47 (13.2)
Serum HCO_3_, median (IQR), mEq/L	25 (22.5–28)	22 (18.3–25.3)	< 0.001^a^	25 (7.0)
Serum HCO_3_ on day 1	25 (22–29)	21 (18–26)	< 0.001^a^	46 (12.9)
Serum lactate, median (IQR), mmol/L	1.8 (1.3–2.3)	2.2 (1.5–3.8)	< 0.001^a^	53 (14.8)
Serum lactate on day 1	1.8 (1.4–2.6)	2.6 (1.5–5.0)	< 0.001^a^	90 (25.2)
PaCO_2_, median (IQR), mm Hg	45 (39–50.4)	42 (36–48.9)	0.02^a^	28 (7.8)
PaO_2_, median (IQR), mm Hg	87 (76–104)	88 (76–111)	0.68	28 (7.8)
PaO_2_:FiO_2_, median (IQR)	139 (107–180)	138 (99–187)	0.72	31 (8.7)
Oxygenation index, median (IQR)	13.8 (7.9–19.5)	14.3 (9.1–23.8)	0.11	68 (19.0)
Fluid overload, median (IQR), %				
Day 1	0.2 (− 0.5 to 2.8)	1 (− 0.3 to 3.1)	0.17	117 (32.8)
Day 2	1.5 (− 0.2 to 3.9)	3.4 (0.8–7.1)	< 0.001	111 (31.1)
Day 3	1.8 (− 0.5 to 5.2)	4.9 (1.8–9.2)	< 0.001	115 (32.2)

*AKI* acute kidney injury, *DP* driving pressure, *FiO*_*2*_ fraction of inspired oxygen, *HCO*_*3*_ bicarbonate, *IQR* interquartile range, *PaCO*_*2*_ partial pressure of carbon dioxide in arterial blood, *PaO*_*2*_ partial pressure of oxygen in arterial blood, *PBW* predicted body weight, *PEEP* positive end-expiratory pressure, *Ppeak* peak inspiratory pressure, *Pplat* plateau pressure, *SD* standard deviation, *VT* tidal volume

^a^*p* < 0.05

^b^Tidal volume ≤ 8 mL/kg PBW and inspiratory plateau pressure ≤ 30 cm H_2_O

**Fig. 2:**
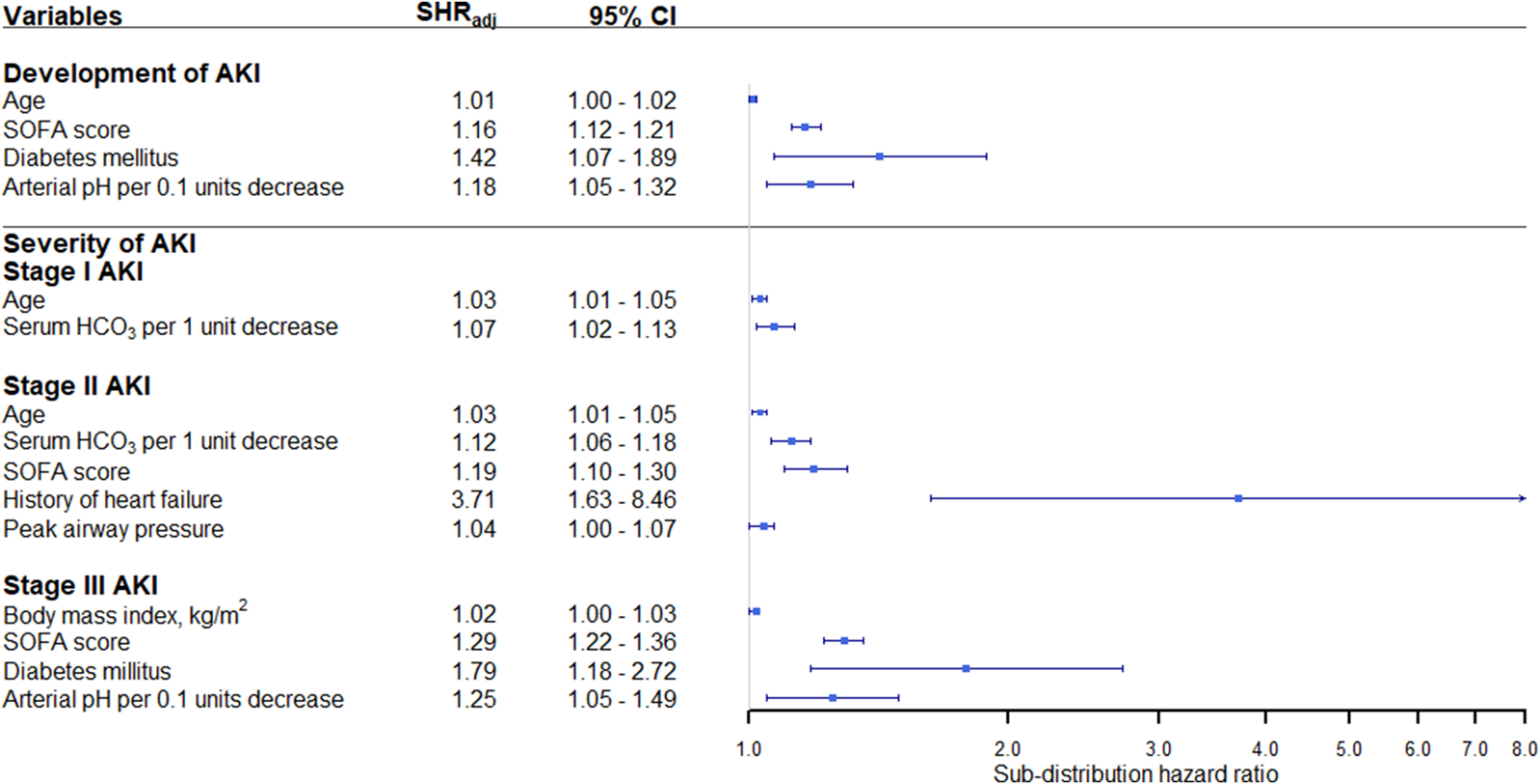
Forest plot showing the results of multivariable competing risk regression analysis for factors associated with development of acute kidney injury and severity of acute kidney injury. The x-axis represents the adjusted subdistribution hazard ratio (SHR_adj_) on a log scale with the reference line (solid vertical line), adjusted subdistribution hazard ratio (square), and 95% confidence interval (whisker)

After adjustment in a multivariable analysis of competing risk model, development of AKI was independently associated with age [SHR 1.01, 95% confidence interval (CI) 1.00–1.02], SOFA score (SHR 1.16, 95%CI 1.12–1.21), a history of DM (SHR 1.42, 95%CI 1.07–1.89), and arterial pH on day 1 of ARDS (SHR per 0.1 units decrease was 1.18, 95%CI 1.05–1.32) (Fig. 2).

In a subgroup analysis of patients without septic shock, age (SHR 1.02, 95%CI 1.00–1.03), SOFA score (SHR 1.18, 95%CI 1.10–1.27), and history of DM (SHR 1.59, 95%CI 1.05–2.41) remained significantly associated with the development of AKI (Additional file 1: Table S9).

### Factors associated with severity of AKI

Severity of AKI was associated with the presence of a higher age, a higher BMI, a high comorbidity index, and the initial severity of illness. Rates of DM, heart failure, active malignancy, and liver disease were significantly higher with worsening severity of AKI. Patients in each stage of AKI had higher extra-pulmonary causes of ARDS, a higher prevalence of septic shock, and a higher prevalence of having received neuromuscular blocking agents, inhaled vasodilators, and recruitment maneuvers. Use of nephrotoxic agents was not associated with development of severity of AKI (Additional file 1: Table S1). Patients also received higher positive end-expiratory pressure (PEEP), peak and mean airway pressure, minute ventilation with higher severity of AKI and these patients had worse oxygenation index, acidosis at the time of onset of AKI, and a higher amount of fluid overload (Additional file 1: Table S4).

In our competing risk regression analysis, a higher age (SHR 1.03, 95%CI 1.01–1.05) and a lower serum HCO_3_ at ARDS onset (SHR per 1 unit decrease was 1.07, 95%CI 1.02–1.13) were independently associated with stage I AKI. Patients with stage II AKI were associated with a higher age (SHR 1.03, 95%CI 1.01–1.05), a lower serum HCO_3_ (SHR per 1 unit decrease was 1.12, 95%CI 1.06–1.18), a higher SOFA score (SHR 1.19, 95%CI 1.10–1.30), history of heart failure (SHR 3.71, 95%CI 1.63–8.46), and a higher average peak airway pressure on day 1–3 of ARDS (SHR 1.04, 95%CI 1.00–1.07). Patients with stage III AKI were associated with a higher BMI (SHR 1.02, 95%CI 1.00–1.03), history of DM (SHR 1.79, 95%CI 1.18–2.72), a higher SOFA score (SHR 1.29, 95%CI 1.22–1.36), and arterial pH on day 1 of ARDS (SHR per 0.1 units decrease was 1.25, 95%CI 1.05–1.49) (Fig. 2).

When we limited our analysis to only patients without septic shock, the stage I AKI was significantly associated with a lower ejection fraction (SHR 0.97, 95%CI 0.93–1.00). The stage II AKI was significantly associated with SOFA score (SHR 1.22, 95%CI 1.09–1.38), history of heart failure (SHR 6.50, 95%CI 2.46–17.19), and a lower serum HCO_3_ (SHR 1.11, 95%CI 1.03–1.19). The stage III AKI was associated with SOFA score (SHR 1.25, 95%CI 1.09–1.43), Charlson comorbidity index (SHR 1.18, 95%CI 1.04–1.32), arterial pH (SHR per 0.1 units decrease was 1.34, 95%CI 1.06–1.70), and inhaled vasodilators (SHR 2.52, 95%CI 1.18–5.39) (Additional file 1: Table S10).

### Renal replacement therapy

A total of 118 patients were found to be in stage III AKI. Of those, 78 (66%) patients underwent RRT. Continuous renal replacement therapy was initiated in 69 (88.5%) patients, and 27 (39.1%) patients were transitioned to intermittent hemodialysis. Intermittent hemodialysis was initiated in 9 (11.5%) patients during their ICU course. Median time to RRT initiation was day 5 of ARDS (IQR 3–8). Among stage III AKI patients, there was no statistical difference in 28-day (55.1% and 55% *p* = 0.99) and 90-day (62.8% and 60% *p* = 0.77) mortality between patients that received RRT and patients that did not.

### AKI and patient outcomes

**Fig. 3:**
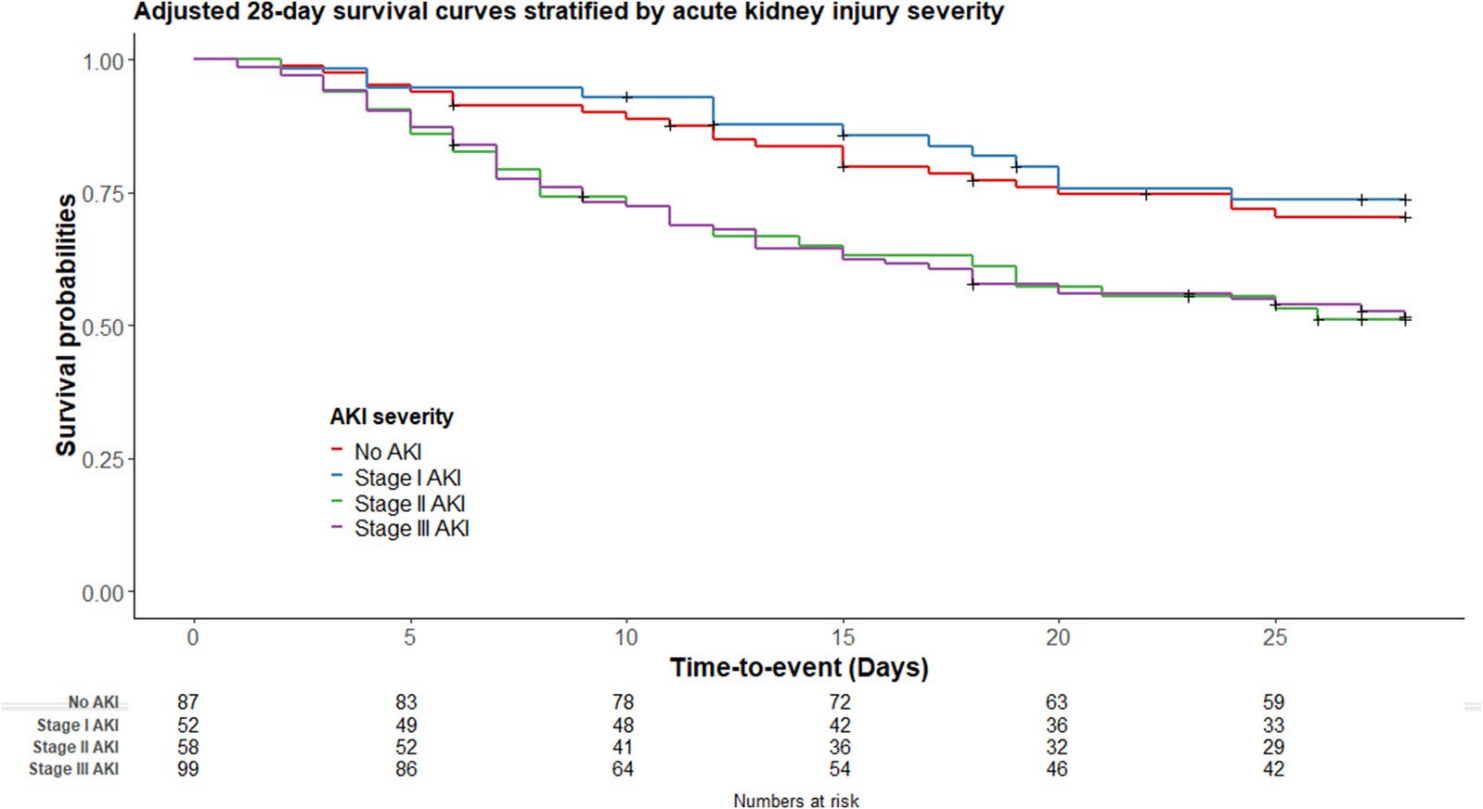
Adjusted 28-day survival curves stratified by severity of acute kidney injury (adjusted for PaO_2_:FiO_2_ on day 1, APACHE III score, and septic shock)

There was a decreased likelihood of unassisted breathing, 28-day survival, and 90-day survival with each stage of AKI severity (Table 3). Patients with stage II and III AKI had a significantly higher risk of death at day 28 and day 90 when compared to patients without AKI. However, patients with stage I AKI did not have a decrease in survival when compared to patients without AKI. After adjustment for PaO_2_:FiO_2_ on day 1, APACHE III score, septic shock, stage II and III AKI remained associated with a lower likelihood of 28-day and 90-day survival. Adjusted hazard ratio of stage II and III AKI at day 28 was 2.00 (95%CI 1.14–3.52, *p* = 0.02) and 1.98 (95%CI 1.19–3.31, *p* = 0.01), respectively. Adjusted hazard ratio of stage II and III AKI at day 90 was 2.44 (95%CI 1.46–4.06, *p* < 0.001) and 2.18 (95%CI 1.35–3.51, *p* = 0.001), respectively. Adjusted 28-day survival curves are shown in Fig. 3.

**Table 3:** Outcomes among patients with and without acute kidney injury

Outcomes	No AKI (113)	Stage I AKI (60)	Stage II AKI (66)	Stage III AKI (118)	*p*
Ventilator-free days to day 28, median (IQR), days	13 (0–20)	12 (0–18)	0 (0–14)^a^	0 (0–0)^a, b^	< 0.001
Duration of mechanical ventilation, median (IQR), days	11 (6–18.3)	12 (8–18.3)	12 (7–20.8)	13 (6–22.8)	0.43
ICU length of stay, median (IQR), days	12 (6.8–17)	13 (9–22)	13 (6.3–20.8)	13 (7–25)	0.58
Hospital length of stay, median (IQR), days	16 (10–24.3)	17 (12.8–26)	20 (11.5–26.8)	19 (8–29)	0.82
ICU mortality (%)	32 (28.6)	17 (28.3)	32 (48.5)	72 (61)^a, b^	< 0.001
Hospital mortality (%)	32 (28.6)	18 (30)	36 (54.5)^a^	73 (61.9)^a, b^	< 0.001
Day 28 mortality (%)	29 (25.7)	19 (31.7)	32 (48.5)^a^	65 (55.1)^a, b^	< 0.001
Day 90 mortality (%)	35 (31)	22 (36.7)	40 (60.6)^a^	73 (61.9)^a, b^	< 0.001

*AKI* acute kidney injury, *ICU* intensive care unit, *IQR* interquartile range

^a^*p* < 0.05 when compared with patients without AKI

^b^*p* < 0.05 when compared with patients with stage I AKI

### Sensitivity analysis

The multivariable competing risk regression analysis with Fine–Gray model on non-imputed data showed that severity of illness, comorbidity, and acidosis had a significantly association with development of AKI. The stage I AKI was associated with age and serum HCO_3_ on day 1 of ARDS. The stage II AKI was associated with age, serum HCO_3_ on day 1, SOFA score, history of heart failure, and average peak airway pressure on days 1–3. The stage III AKI was associated with SOFA score, arterial pH on day 1, BMI, and comorbidity index (Additional file 1: Table S11).

## Discussion

Our study found that two-third of ARDS patients developed AKI during their ICU course and almost half of these patients deteriorated to stage III AKI, based on the KDIGO classification. In ARDS patients, age, presence of DM and heart failure, worsening acidosis on day 1 of ARDS, a higher severity of illness score (SOFA and APACHE III) are strongly associated with the development of AKI. Ventilator-specific variable, peak airway pressure, was associated with the severity of AKI, especially stage II AKI. These findings held true when we limited our model to non-septic patients.

Several studies have shown that older age is associated with the development of AKI among hospitalized patients [7, 26, 27]. Our cohort finds similar trends, and the development of AKI was strongly associated with age of the patients. Similar to findings by Hsu et al. and Hoste et al., our study found that DM increases the risk of AKI, independent of GFR [28, 29]. Since diabetic kidneys have a higher susceptibility to ischemic–hypoxic insults [30], it is plausible that ARDS patients with DM had a greater risk of the development of AKI. A history of cardiac disease was found to be independently associated with severity of AKI that was well described as a risk factor for AKI [7, 26]. A higher severity of illness (SOFA score or APACHE III score) had a significant association with the development and severity of AKI in our study. The risk of hemodynamic derangements associated with worsening severity of illness scores likely is the reason for these findings. Acidosis on day 1 of ARDS was strongly associated with the development and severity of AKI in our cohort of patients. This effect remained significant even after adjustment for the severity of illness. Hemodynamic alterations and cellular adverse effects associated with acidosis can explain the increased risk of AKI associated with worsening pH [10]. Similar to other recent studies, our study showed that obesity was associated with the development of AKI and the severity of AKI in ARDS patients [31, 32]. This effect might be driven by the higher rates of pro-inflammatory markers and acute phase proteins in obese patients [32].

Over the years, many studies have postulated that mechanical ventilation might have a significant part in the deleterious effect on the kidneys in ARDS patients due to multiple mechanisms such as cardiovascular, intrarenal, and hormonal changes in these patients [8, 33, 34]. Lung protective strategies have been postulated to decrease renal failure in critically ill patients [3, 4]. The ARMA trial showed that patients who had a low tidal volume and limited plateau pressure had a greater number of days without renal failure [35]. Although these studies reported a higher number of renal failure free days, they did not directly evaluate association of AKI with mechanical ventilation settings. Although several mechanical ventilation settings had a significantly association with AKI in univariable model, in the era of lung protective ventilation, we found only peak airway pressure was associated with severity of AKI. Peak airway pressure is a secondary parameter that is associated with tidal volume, external and intrinsic PEEP, and respiratory system mechanics. Although low tidal volume ventilation, limited plateau pressure, and PEEP level were not associated with the development of AKI in our multivariable model, these parameters should be controlled in order to maintain adherence to lung protective strategies. We performed a subgroup analysis on patients that did not have septic shock and were thus not at risk of developing AKI due to hemodynamic derangements as part of a multi-organ failure. Ventilator-specific variables remained nonsignificant in this cohort of patients.

Our study found that fluid overload in the first 72 h was associated with severity of AKI. Several studies reported the association between positive fluid balance and development of AKI and AKI severity [36–38]. Fluid overload can directly impact on kidney functions via increased venous pressure and interstitial edema [39]. However, we did not have information about the association between fluid balance on day 1 of ARDS and development of AKI. Interestingly, use of inhaled vasodilators was an independent risk factor for severity of AKI in patients without septic shock. This result was in line with the study of Ruan SY et al. that showed that patients with moderate to severe ARDS who received inhaled nitric oxide had a significantly higher risk of needing RRT [40, 41]. Among critically patients, there is a stepwise increase in mortality with increasing AKI severity, particularly KDIGO stage II and III [27]. The multinational AKI-EPI study showed that patients with stage I AKI did not have a higher risk of mortality compared to patients without AKI [29]. These findings were emulated in our cohort of ARDS patients and the risk of death increased significantly with stage II and stage III AKI. Because 98% of patients with stage I AKI had complete renal recovery, patients with stage I AKI did not have a higher mortality compared to patients without AKI.

We acknowledge a number of limitations of the current study, which are in part due to the retrospective study design. We did not have initial ventilator settings in approximately ten percent of the patients; greater prevalence of this was observed in patients who were transferred from an outside hospital. We addressed this problem by using a multiple imputation method. Post hoc power analysis for binary logistic regression using covariate binomial distribution found that our study had 62% power. Since half of the study population did not have a baseline SCr, we addressed this problem using estimate SCr by back calculation with the MDRD equation. Therefore, the proportion of AKI may be overestimated, and we may have misclassified the severity of AKI [21, 42, 43]. But we have adequately addressed these limitations by using standardized, validated definitions, imputation methods that have been validated in the literature and by using exhaustive multivariable regression models and subgroup analysis to minimize any potential biases or confounding in our results. Our study also used a validated definition for AKI [19] which used both SCr and urine volume parameters. In addition, we excluded the patients who had AKI or chronic kidney disease prior to onset of ARDS.

To our knowledge, this is the first study to explore a number of hitherto overlooked confounders [comorbidities, initial severity of illness, progression of disease (need for vasopressors, development of sepsis) and ICU specific interventions (ventilator settings, rescue therapies)] associated with the development of AKI in ARDS patient.

## Conclusions

In conclusion, similar to other critically ill patients, age, a history of DM, initial severity of illness, and severity of acidosis at the time of ARDS diagnosis were associated with the development of AKI in ARDS patients. Ventilator-specific variables had no impact on the development of AKI. Severity of AKI was further associated with the BMI, history of heart failure, and peak airway pressure in our cohort.

## Additional file

**Additional file 1.** Supplementary tables and figures.

## Data Availability

The datasets used and/or analyzed during the current study are available from the corresponding author on reasonable request.

## Abbreviations

AKI: acute kidney injury
APACHE: acute physiology, age, chronic health evaluation
ARDS: acute respiratory distress syndrome
BMI: body mass index
CI: confidence interval
DM: diabetes mellitus
GFR: glomerular filtration rate
ICU: intensive care unit
IQR: interquartile range
KDIGO: Kidney Disease Improving Global Outcomes
MDRD: Modification of Diet in Renal Disease
PaCO_2_: partial pressure of carbon dioxide
RRT: renal replacement therapy
SOFA: sequential organ failure assessment
SCr: serum creatinine
SHRs: subdistribution hazard ratios
VILI: ventilator-induced lung injury
