# Correction to: Factors associated with acute kidney injury in acute respiratory distress syndrome

**DOI:** 10.1186/s13613-019-0558-z

**Published:** 2019-07-23

**Authors:** Anupol Panitchote, Omar Mehkri, Andrei Hastings, Tarik Hanane, Sevag Demirjian, Heather Torbic, Eduardo Mireles-Cabodevila, Sudhir Krishnan, Abhijit Duggal

**Affiliations:** 1 Department of Critical Care, Respiratory Institute, Cleveland Clinic, Cleveland, OH USA; 2 Division of Critical Care Medicine, Department of Medicine, Faculty of Medicine, Khon Kaen University, Khon Kaen, Thailand; 3 Department of Nephrology, Cleveland Clinic, Cleveland, OH USA; 4 Department of Pharmacology, Cleveland Clinic, Cleveland, OH USA

## Correction to: Ann Intensive Care (2019) 9:74 10.1186/s13613-019-0552-5

After publication of the original article [1], we were notified that an author’s name has been incorrectly spelled. Andrei Hasting should be replaced with Andrei Hastings.

The original article has been corrected.

The publisher apologies for the inconvenience.
