# Supplementary material for: Factors associated with acute kidney injury in acute respiratory distress syndrome
Source: Ann Intensive Care. 2019 Jul 1;9:74. doi: 10.1186/s13613-019-0552-5 (PMC6603088; doi:10.1186/s13613-019-0552-5)
Supplement: Supplementary file 1 — Additional file 1. Supplementary tables and figures. [file 13613_2019_552_MOESM1_ESM.docx]

**Supplementary Digital Content**

**Factors associated with acute kidney injury in acute respiratory distress syndrome**

Anupol Panitchote, MD; Omar Mehkri, MD; Andrei Hasting, MD; Tarik Hanane, MD; Sevag Demirjian, MD; Heather Torbic PharmD; Eduardo Mireles- Cabodevila, MD; Sudhir Krishnan, MD; Abhijit Duggal, MD, MPH, MSc

**Appendix 1.** Imputation process

**Table S1.** Baseline Characteristics by Severity of Acute Kidney Injury

**Table S2.** Ventilator Settings Averaged on Day 1 to 3, Arterial Blood Gas, and Fluid Overload by Severity of Acute Kidney Injury

**Table S3.** Univariable Analysis of Subdistribution Hazard Ratios for Factors Associated with the Development of Acute Kidney Injury in Patients with Acute Respiratory Distress Syndrome

**Table S4.** Univariable Analysis of Subdistribution Hazard Ratios for Factors Associated with the Development of Severity of Acute Kidney Injury in Patients with Acute Respiratory Distress Syndrome

**Table S5.** Baseline Characteristics by Acute Kidney Injury in Acute Respiratory Distress Syndrome Patients without Septic Shock

**Table S6.** Ventilator Settings Averaged on Day 1 to 3, Arterial Blood Gas, and Fluid Overload in Acute Respiratory Distress Syndrome Patients without Septic Shock by Acute Kidney Injury

**Table S7**. Baseline Characteristics by Severity of Acute Kidney Injury in Acute Respiratory Distress Syndrome Patients without Septic Shock

**Table S8.** Ventilator Settings Averaged on Day 1 to 3, Arterial Blood Gas, and Fluid Overload in Acute Respiratory Distress Syndrome Patients without Septic Shock by Severity of Acute Kidney Injury

**Table S9**. Multivariable Competing Risk Regression for Factors Associated with the Development of Acute Kidney Injury in ARDS Patients without Septic Shock

**Table S10.** Multivariable Competing Risk Regression for Factors Associated with the Development of Severity of Acute Kidney Injury in Acute Respiratory Distress Syndrome Patients without Septic Shock

**Table S11.** Sensitivity Analysis of Non-Imputed Data Using Multivariable Competing Risk Regression for Factors Associated with the Development of Acute Kidney Injury and Severity of Acute Kidney Injury in Acute Respiratory Distress Syndrome Patients

**Figure S1.** Cumulative incidence curves for development acute kidney injury (AKI) by AKI severities.

**Appendix 1. Imputation process**

The missing data was created using multiple imputation by chained equations and analyzed 50 imputed data sets in order to complete a logistic regression models [1]. The imputation process included variables that were cooperated into regression models and also included outcomes variables [2]. Calculations were performed using R version 3.6.0 using automatic predictor selection tool of the mice 3.3.0 package [3]. The procedure assumes the missing data to be missing at random. The model estimates and standard errors were combined into a single set of results using Rubin’s rules.

**Table S1. Baseline Characteristics by Severity of Acute Kidney Injury**

| Characteristics | No AKI  (113) | | Stage I AKI  (60) | | Stage II AKI  (66) | | Stage III AKI (118) | | *p* | |
| --- | --- | --- | --- | --- | --- | --- | --- | --- | --- | --- |
| Age, mean (SD), years | 50.2 | (15.8) | 59.2 | (16.6)^a^ | 56.3 | (14.3) | 50.7 | (15.3)^b^ | < | 0.001 |
| Male sex, n (%) | 54 | (47.8) | 31 | (51.7) | 38 | (57.6) | 68 | (57.6) |  | 0.42 |
| BMI, median (IQR), kg/m^2^ | 29.7 | (24.6-35.5) | 27.1 | (23.2-34.7) | 29 | (24.6-35.8) | 32.6 | (25.6-41.6)^b^ |  | 0.01 |
| Race, n (%)  White  Black or African American | 88  20 | (77.9)  (17.7) | 42  16 | (70)  (26.7) | 49  16 | (74.2)  (24.2) | 79  26 | (66.9)  (22) |  | 0.29  0.54 |
| SOFA, mean (SD), points | 9 | (2.6) | 10.1 | (2.9) | 11.2 | (3.2)^a^ | 13.1 | (3.6)^a, b, c^ | < | 0.001 |
| Non-renal SOFA, mean (SD), points | 8.9 | (2.6) | 9.5 | (2.7) | 10.4 | (2.8) ^a^ | 11.4 | (3) ^a, b^ | < | 0.001 |
| APACHE III, mean (SD), points | 95 | (26) | 101 | (29) | 110 | (25)^a^ | 125 | (33)^a, b, c^ | < | 0.001 |
| Charlson comorbidities index, median (IQR), points | 2 | (0-4) | 3 | (1.8-6) | 3.5 | (1.3-5)^a^ | 3 | (1-5) |  | 0.01 |
| Comorbidities, n (%)  Chronic lung diseases  Diabetes  Active malignancies  Liver disease  Heart failure  Recent surgery within 3 months. | 44  23  18  5  5  4 | (38.9)  (20.4)  (15.9)  (4.4)  (4.4)  (3.5) | 20  14  10  2  7  3 | (33.3)  (23.3)  (16.7)  (3.3)  (11.7)  (5) | 23  20  16  9  8  3 | (34.8)  (30.3)  (24.2)  (13.6)  (12.1)  (4.5) | 31  38  33  17  7  4 | (26.3)  (32.2)  (28)  (14.4)  (5.9)  (3.4) |  | 0.23  0.18  0.11  0.01  0.13  0.89 |
| Cause of ARDS, n (%)  Pneumonia  Aspiration  Non-pulmonary sepsis  Pancreatitis | 91  22  3  6 | (80.5)  (19.5)  (2.7)  (5.3) | 51  14  7  2 | (85)  (23.3)  (11.7)  (3.3) | 55  13  3  3 | (83.3)  (19.7)  (4.5)  (4.5) | 98  16  11  3 | (83.1)  (13.6)  (9.3)  (2.5) |  | 0.89  0.39  0.06  0.74 |
| Echocardiographic findings  EF, median (IQR), %  RVSP, median (IQR), mm Hg | 60  38 | (55-65)  (31-48) | 57  37 | (55-62)  (27-47) | 61  41 | (55-65)  (32.5-46.4) | 60  41 | (53-65)  (32-52) |  | 0.34  0.36 |
| Septic shock | 37 | (32.7) | 27 | (45) | 31 | (47) | 84 | (71.2)^a, b, c^ | < | 0.001 |
| Nephrotoxic agents, n (%)  Antimicrobial agents  Contrast agents  ACEI  Calcineurin inhibitors  NSAIDs | 103  34  15  5  4 | (91.2)  (30.1)  (13.3)  (4.4)  (3.5) | 53  19  10  2  0 | (89.8)  (31.7)  (16.7)  (3.3)  (0) | 53  17  10  5  2 | (81.5)  (25.8)  (15.2)  (7.6)  (3) | 89  28  7  3  2 | (83.2)  (23.9)  (5.9)  (2.5)  (1.7) |  | 0.17  0.63  0.10  0.44  0.48 |
| Number of nephrotoxic agents, median (IQR), count | 2 | (1-3) | 2 | (1-3) | 2 | (1-3) | 2 | (1-3) |  | 0.82 |
| Rescue therapies (%)  Continuous NMBA  Inhaled vasodilators  Prone positioning  ECMO  Recruitment maneuvers  HFOV | 37  21  13  5  4  2 | (32.7)  (18.6)  (11.5)  (4.4)  (3.5)  (1.8) | 13  8  6  2  2  2 | (21.7)  (13.3)  (10)  (3.3)  (3.3)  (3.3) | 24  17  6  0  4  2 | (36.4)  (25.8)  (9.1)  (0)  (6.1)  (3) | 62  39  22  5  14  7 | (52.5)^a, b^  (33.1)  (18.6)  (4.2)  (11.9)  (5.9) | < | 0.001  0.01  0.18  0.38  0.06  0.43 |
| Known baseline SCr, n (%) | 57 | (50.4) | 34 | (56.7) | 46 | (69.7) | 65 | (55.1) |  | 0.09 |
| Baseline SCr, mean (SD), mg/dL | 0.79 | (0.18) | 0.82 | (0.21) | 0.83 | (0.21) | 0.81 | (0.18) |  | 0.84 |
| eGFR, median (IQR), mL/min per 1.73 m^2^ | 98.2 | (80.7-111.8) | 81.8 | (73-110.2) | 95.6 | (80.7-109.2) | 97.1 | (84.6-115.2) |  | 0.27 |
| Time from ARDS diagnosis to hospital admission (IQR), days | 0 | (-3 to 1) | 0 | (-0.3 to 2) | 0.5 | (-1 to 4) | 0 | (-2 to 1) |  | 0.08 |

ACEI = angiotensin converting enzyme inhibitors, AKI = acute kidney injury, APACHE = acute physiology, age, chronic health evaluation, ARDS = acute respiratory distress syndrome, BMI = body mass index, ECMO = extracorporeal membrane oxygenation, EF = ejection fraction, eGFR = estimated glomerular filtration rate, HFOV = high frequency oscillatory ventilation, IQR = interquartile range, NMBA = neuromuscular blocking agents, NSAIDs = non-steroidal anti-inflammatory drugs, RVSP = right ventricular systolic pressure, SCr = serum creatinine, SD = standard deviation, SOFA = sequential organ failure assessment.

^a^p < 0.05 when compared with patients without AKI.

^b^p < 0.05 when compared with patients with stage I AKI.

^c^p < 0.05 when compared with patients with stage II AKI.

**Table S2.** **Ventilator Settings Averaged on Day 1 to 3, Arterial Blood Gas, and Fluid Overload by Severity of Acute Kidney Injury**

| Ventilator settings | No AKI  (113) | | Stage I AKI  (60) | | Stage II AKI  (66) | | Stage III AKI (118) | | *p* | |
| --- | --- | --- | --- | --- | --- | --- | --- | --- | --- | --- |
| Spontaneous VT, median (IQR), mL | 451 | (401-502) | 475 | (414-534) | 515 | (422-556) | 489 | (430-548) |  | 0.03 |
| VT, median (IQR), (mL/kg PBW) | 7.4 | (6.7-8.6) | 7.5 | (6.6-8.2) | 7.5 | (7.1-8.3) | 7.6 | (6.8-8.6) |  | 0.87 |
| PEEP, median (IQR), cm H_2_O | 10.7 | (8-13.3) | 9.3 | (7.3-13) | 9.8 | (8-12) | 12 | (8.8-14.7) |  | 0.01 |
| FiO_2_, median (IQR) | 0.67 | (0.54-0.8) | 0.7 | (0.57-0.8) | 0.65 | (0.53-0.85) | 0.73 | (0.57-0.9) |  | 0.31 |
| Pplat, median (IQR), cm H_2_O | 27 | (23-31) | 25.5 | (21-31.3) | 27.5 | (22.8-33.3) | 27.5 | (23.3-34.6) |  | 0.46 |
| Pplat > 30 cm H_2_O (%) | 18 | (30.5) | 11 | (32.4) | 14 | (35) | 26 | (39.4) |  | 0.76 |
| DP, median (IQR), cm H_2_O | 15 | (11.3-18.5) | 13.8 | (11.4-18.4) | 15 | (12.5-20.4) | 14.5 | (12-18.6) |  | 0.75 |
| Mean airway pressure, median (IQR), cm H_2_O | 17.3 | (14.3-20.7) | 17.8 | (14 -19.8) | 16.2 | (14.3-21.5) | 18.7 | (15.3-22) |  | 0.12 |
| Ppeak, mean (SD), cm H_2_O | 29.6 | (6.5) | 29.6 | (5.5) | 31.7 | (8.1) | 31.7 | (7.6) |  | 0.09 |
| Minute ventilation, median (IQR), L/min | 10.7 | (8.5-11.9) | 11 | (9.2-12.6) | 11 | (9.1-13.9) | 11.6 | (10.1-13.3)^a^ |  | 0.02 |
| Lung protective ventilation, n (%)^c^ | 26 | (35.1) | 18 | (41.9) | 18 | (36.7) | 28 | (32.9) |  | 0.80 |
| Arterial blood gas |  |  |  |  |  |  |  |  |  |  |
| Arterial pH, median (IQR) | 7.39 | (7.34-7.43) | 7.38 | (7.33-7.42) | 7.35 | (7.3-7.41) | 7.34 | (7.28-7.38)^a, b^ | < | 0.001 |
| Arterial pH on day 1 | 7.38 | (7.32-7.42) | 7.36 | (7.31-7.44) | 7.35 | (7.3- 7.41) | 7.33 | (7.27-7.39)^a^ |  | 0.003 |
| Serum HCO_3_, median (IQR), mEq/L | 25 | (22.5-28) | 23.5 | (19.8-26.7)^a^ | 23 | (19.8-25.4)^a^ | 20.3 | (17-24.3)^a, b^ | < | 0.001 |
| Serum HCO_3_ on day 1 | 25 | (22 -29) | 22.5 | (19-27.3) | 22 | (18-26)^a^ | 21 | (16-24)^a^ | < | 0.001 |
| Serum lactate, median (IQR), mmol/L | 1.8 | (1.3-2.3) | 1.8 | (1.4-3.0) | 2.0 | (1.5-3.4) | 2.5 | (1.5-5.4)^a^ | < | 0.001 |
| Serum lactate on day 1 | 1.8 | (1.4-2.6) | 2.4 | (1.6-3.7) | 2.0 | (1.4-4.2) | 3.0 | (1.7-7.2)^a^ | < | 0.001 |
| PaCO_2_, median (IQR), mm Hg | 45 | (39-50.4) | 42 | (38-48.3) | 42 | (35.8-50) | 42.3 | (36-49.7) |  | 0.12 |
| PaO_2_, median (IQR), mm Hg | 87 | (76-104) | 89 | (75-111) | 87 | (76-111) | 86 | (77-109) |  | 0.95 |
| PaO_2_:FiO_2_, median (IQR) | 139 | (107-180) | 143 | (113-188) | 141 | (99-178) | 137 | (94-196) |  | 0.84 |
| Oxygenation index, median (IQR) | 13.8 | (7.9-19.5) | 14.4 | (9.5-19.5) | 13 | (8.7-21.9) | 15.1 | (9.3-26.1) |  | 0.26 |
| Fluid overload, median (IQR), %  Day 1  Day 2  Day 3 | 0.2  1.5  1.8 | (-0.5 to 2.8)  (-0.2 to 3.9)  (-0.5 to 5.2) | 0.3  2.5  4 | (-0.7 to 3.2)  (0.2 to 6.3)  (0.9 to 8.7) | 1.9  3.4  5.7 | (-0.1 to 4.3)  (1.4 to 7.3)  (2.1 to 9.6) | 1.0  3.5  5.2 | (-0.1 to 2.7)  (1.1 to 7.9)  (2.3 to 9.3) | < | 0.26  0.002  0.001 |

AKI = acute kidney injury, DP = driving pressure, FiO_2_ = fraction of inspired oxygen, HCO_3_ = bicarbonate, IQR = interquartile range, PaCO_2_ = partial pressure of carbon dioxide in arterial blood, PaO_2_ = partial pressure of oxygen in arterial blood, PBW = predicted body weight, PEEP = positive end-expiratory pressure, Ppeak = peak inspiratory pressure, Pplat = plateau pressure, SD = standard deviation, VT = tidal volume.

^a^p < 0.05 when compared with patients without AKI.

^b^p < 0.05 when compared with patients with stage I AKI.

^c^Tidal volume ≤ 8 mL/kg PBW and inspiratory plateau pressure ≤ 30 cm H_2_O

**Table S3. Univariable Analysis of Subdistribution Hazard Ratios for Factors Associated with the Development of Acute Kidney Injury in Patients with Acute Respiratory Distress Syndrome**

| Variable | Competing risk with Fine-Gray model  (No AKI vs AKI) | | | |
| --- | --- | --- | --- | --- |
|  | **Subdistribution hazard ratio** | **95% Confidence interval** | ***p*** | |
| Age, year^a^ | 1.01 | 1.00-1.02 |  | 0.02 |
| Male sex | 1.19 | 0.93-1.54 |  | 0.17 |
| BMI (kg/m^2^)^a^  Non-obese (BMI < 30)  Class I obesity (BMI 30-34.9)  Class II obesity (BMI 35-39.9)  Class III obesity (BMI ≥ 40) | 1.00  Ref  0.98  1.14  1.10 | 0.99-1.01  Ref  0.70-1.37  0.73-1.77  0.79-1.51 |  | 0.78  Ref  0.88  0.56  0.58 |
| Race  White  Black or African American | 0.77  1.30 | 0.59-1.01  0.97-1.75 |  | 0.06  0.08 |
| SOFA score^a^ | 1.17 | 1.13-1.21 | < | 0.001 |
| Non-renal SOFA score^a^ | 1.13 | 1.08-1.18 | < | 0.001 |
| APACHE III score^a^ | 1.02 | 1.01-1.02 | < | 0.001 |
| Charlson comorbidities index^a^ | 1.08 | 1.03-1.13 |  | 0.002 |
| Comorbidities  Chronic lung diseases  Diabetes  Active malignancies  Liver disease  Heart failure  Recent surgery within 3 months. | 0.82  1.23  1.36  1.41  1.36  1.13 | 0.62-1.07  0.93-1.62  1.01-1.83  0.95-2.09  0.88-2.11  0.60-2.13 |  | 0.15  0.15  0.04  0.09  0.17  0.70 |
| Cause of ARDS  Pneumonia  Aspiration  Non-pulmonary sepsis  Pancreatitis | 1.10  0.94  1.50  0.86 | 0.78-1.55  0.67-1.31  0.95-2.36  0.43-1.75 |  | 0.58  0.70  0.09  0.69 |
| Echocardiographic findings^a^  EF (%)  RVSP, mm Hg | 0.99  1.00 | 0.98-1.00  0.99-1.01 |  | 0.07  0.48 |
| Septic shock | 1.78 | 1.38-2.30 | < | 0.001 |
| Nephrotoxic agents  Antimicrobial agents  Contrast agents  ACEI  Calcineurin inhibitors  NSAIDs | 0.63  0.89  0.86  0.92  0.53 | 0.44-0.90  0.67-1.19  0.57-1.29  0.49-1.73  0.20-1.43 |  | 0.01  0.45  0.46  0.79  0.21 |
| Number of nephrotoxic agents | 0.96 | 0.87-1.07 |  | 0.50 |
| Rescue therapies  Continuous NMBA  Inhaled vasodilators  Prone positioning  ECMO  Recruitment maneuvers  HFOV | 1.13  1.09  1.06  0.78  1.18  1.44 | 0.88-1.46  0.82-1.44  0.74-1.52  0.37-1.66  0.74-1.86  0.78-2.63 |  | 0.34  0.57  0.75  0.52  0.48  0.24 |
| Ventilator settings (average on day 1-3)  Tidal volume, mL^a^  Tidal volume, mL/kg of PBW^a^  PEEP^a^  PEEP ≥ 16 cm H_2_O  FiO_2_^a^  Plateau pressure, cm H_2_O^a^  Plateau pressure > 30 cm H_2_O  Driving pressure, cm H_2_O^a^  Mean airway pressure, cm H_2_O^a^  Peak airway pressure, cm H_2_O^a^  Minute ventilation, L/min^a^ | 1.00  0.98  1.02  1.34  1.38  1.01  1.03  1.00  1.03  1.02  1.09 | 1.00-1.00  0.90-1.07  0.98-1.05  0.93-1.94  0.69-2.76  0.98-1.03  0.73-1.45  0.97-1.03  1.00-1.05  1.00-1.04  1.04-1.15 | < | 0.08  0.64  0.34  0.12  0.36  0.71  0.88  0.96  0.049  0.14  0.001 |
| Arterial blood gases (average on day 1-3)  Arterial (per 0.1 points decrease)  Arterial pH on day 1 (per 0.1 points decrease)  Serum HCO_3_ (per 1 point decrease)  Serum HCO_3_ on day 1 (per 1 point decrease)  PaCO_2_, mm Hg^a^  PaO_2_, mm Hg^a^ | 1.48  1.27  1.10  1.07  0.98  1.00 | 1.26-1.73  1.13-1.43  1.07-1.13  1.05-1.10  0.97-1.00  1.00-1.01 | <  <  <  < | 0.001  0.001  0.001  0.001  0.02  0.57 |
| PaO_2_:FiO_2_ ratio^a^ | 1.00 | 1.00-1.00 |  | 0.90 |
| Oxygenation index^a^ | 1.01 | 1.00-1.02 |  | 0.02 |
| Fluid overload, median (IQR), %  Day 1  Day 2  Day 3 | 1.07  1.07  1.04 | 1.01-1.13  1.03-1.10  1.02-1.07 | <  < | 0.01  0.001  0.001 |

ACEI = angiotensin converting enzyme inhibitors, APACHE = Acute Physiology, Age, Chronic Health Evaluation, BMI = body mass index, CI = confidence interval, ECMO = extracorporeal membrane oxygenation, FiO_2_ = fractional of inspired oxygen, HFOV = high frequency oscillatory ventilation, NMBA = neuromuscular blocking agents, NSAIDs = non-steroidal anti-inflammatory drugs, PaCO_2_ = partial pressure of carbon dioxide in arterial blood, PaO_2_ = partial pressure of oxygen in arterial blood, PBW = predicted body weight, PEEP = positive end-expiratory pressure, RVSP = right ventricular systolic pressure, SOFA = sequential organ failure assessment.

^a^per 1 point increase

**Table S4. Univariable Analysis of Subdistribution Hazard Ratios for Factors Associated with the Development of Severity of Acute Kidney Injury in Patients with Acute Respiratory Distress Syndrome**

| Variable | Competing risk with Fine-Gray model | | | | | |
| --- | --- | --- | --- | --- | --- | --- |
|  | **Stage I AKI** | | **Stage II AKI** | | **Stage III AKI** | |
|  | **SHR** | **95% CI** | **SHR** | **95% CI** | **SHR** | **95% CI** |
| Age, year^a^ | 1.03 | 1.01 to 1.05 | 1.02 | 1.01 to 1.04 | 1.00 | 0.99 to 1.01 |
| Male sex^a^ | 1.20 | 0.72 to 1.99 | 1.30 | 0.80 to 2.12 | 1.29 | 0.90 to 1.86 |
| BMI (kg/m^2^)^a^  Non-obese (BMI < 30)  Class I obesity (BMI 30-34.9)  Class II obesity (BMI 35-39.9)  Class III obesity (BMI ≥ 40) | 0.99  Ref  0.89  1.12  0.68 | 0.96 to 1.02  Ref  0.46 to 1.72  0.50 to 2.53  0.30 to 1.54 | 1.01  Ref  0.87  0.81  1.11 | 0.98 to 1.03  Ref  0.45 to 1.66  0.29 to 2.28  0.60 to 2.05 | 1.01  Ref  1.09  1.37  1.60 | 1.00 to 1.02  Ref  0.67 to 1.79  0.74 to 2.53  1.03 to 2.48 |
| Race  White  Black or African American | 0.71  1.53 | 0.41 to 1.24  0.86 to 2.72 | 0.83  1.38 | 0.48 to 1.45  0.78 to 2.42 | 0.68  1.28 | 0.46 to 1.00  0.83 to 1.98 |
| SOFA score^a^ | 1.13 | 1.04 to 1.24 | 1.23 | 1.14 to 1.33 | 1.29 | 1.22 to 1.36 |
| Non-renal SOFA score^a^ | 1.08 | 0.98 to 1.19 | 1.17 | 1.07 to 1.27 | 1.22 | 1.15 to1.29 |
| APACHE III score^a^ | 1.01 | 1.00 to 1.02 | 1.02 | 1.01 to 1.03 | 1.02 | 1.02 to 1.03 |
| Charlson comorbidities index^a^ | 1.12 | 1.02 to 1.24 | 1.13 | 1.03 to 1.23 | 1.08 | 1.01 to 1.16 |
| Comorbidities  Chronic lung diseases  Diabetes  Active malignancies  Liver disease  Heart failure  Recent surgery within 3 months. | 0.80  1.10  1.10  0.78  2.05  1.15 | 0.47 to 1.38  0.61 to 2.01  0.56 to 2.17  0.19 to 3.21  0.93 to 4.51  0.36 to 3.67 | 0.88  1.43  1.51  2.33  2.26  1.47 | 0.53 to 1.46  0.84 to 2.42  0.86 to 2.65  1.15 to 4.70  1.08 to 4.74  0.46 to 4.69 | 0.68  1.55  1.62  1.84  1.09  1.03 | 0.45 to 1.03  1.05 to 2.28  1.09 to 2.43  1.10 to 3.09  0.51 to 2.33  0.38 to 2.79 |
| Cause of ARDS  Pneumonia  Aspiration  Non-pulmonary sepsis  Pancreatitis | 1.32  1.16  2.48  0.67 | 0.65 to 2.68  0.62 to 2.14  1.07 to 5.79  0.16 to 2.74 | 1.14  1.06  1.22  0.96 | 0.59 to 2.17  0.58 to 1.94  0.38 to 3.89  0.30 to 3.05 | 1.09  0.68  2.08  0.68 | 0.68 to 1.77  0.40 to 1.16  1.11 to 3.89  0.40 to 1.16 |
| Echocardiographic findings^a^  EF (%)  RVSP, mm Hg | 0.98  1.00 | 0.95 to 1.01  0.97 to 1.02 | 1.00  1.01 | 0.97 to 1.02  0.99 to 1.03 | 0.98  1.01 | 0.96 to 1.00  1.00 to 1.03 |
| Septic shock | 1.59 | 0.95 to 2.66 | 1.63 | 1.00 to 2.64 | 3.05 | 2.04 to 4.55 |
| Nephrotoxic agents  Antimicrobial agents  Contrast agents  ACEI  Calcineurin inhibitors  NSAIDs | 0.81  1.04  1.14  0.77  - | 0.35 to 1.89  0.60 to 1.80  0.56 to 2.32  0.19 to 3.17  - | 0.50  0.84  1.04  1.54  0.77 | 0.26 to 0.93  0.49 to 1.46  0.53 to 2.04  0.62 to 3.84  0.19 to 3.16 | 0.54  0.80  0.52  0.62  0.56 | 0.33 to 0.91  0.52 to 1.23  0.24 to 1.13  0.20 to 1.96  0.14 to 2.25 |
| Number of nephrotoxic agents | 0.96 | 0.76 to 1.21 | 0.92 | 0.74 to 1.15 | 1.02 | 0.87 to 1.20 |
| Rescue therapies  Continuous NMBA  Inhaled vasodilators  Prone positioning  ECMO  Recruitment maneuvers  HFOV | 0.64  0.70  0.88  0.74  0.82  1.35 | 0.34 to 1.18  0.33 to 1.48  0.38 to 2.05  0.18 to 3.04  0.20 to 3.37  0.33 to 5.54 | 1.15  1.25  0.81  -  1.32  1.46 | 0.69 to 1.89  0.72 to 2.16  0.35 to 1.88  -  0.48 to 3.64  0.36 to 5.97 | 1.65  1.53  1.38  1.05  1.85  2.13 | 1.15 to 2.37  1.04 to 2.25  0.87 to 2.20  0.43 to 2.56  1.06 to 3.24  0.99 to 4.59 |
| Ventilator settings (average on day 1-3)  Tidal volume, mL^a^  Tidal volume, mL/kg of PBW^a^  PEEP^a^  PEEP ≥ 16 cm H_2_O  FiO_2_^a^  Plateau pressure, cm H_2_O^a^  Plateau pressure > 30 cm H_2_O  Driving pressure, cm H_2_O^a^  Mean airway pressure, cm H_2_O^a^  Peak airway pressure, cm H_2_O^a^  Minute ventilation, L/min^a^ | 1.00  0.94  0.97  1.43  1.06  0.99  0.98  1.00  1.00  1.00  1.06 | 1.00 to 1.00  0.78 to 1.12  0.90 to 1.04  0.65 to 3.16  0.25 to 4.39  0.94 to 1.04  0.48 to 2.01  0.94 to 1.06  0.94 to 1.07  0.95 to 1.04  0.95 to 1.17 | 1.00  0.97  0.98  0.96  1.43  1.02  1.12  1.03  1.03  1.03  1.09 | 1.00 to 1.01  0.82 to 1.15  0.91 to 1.05  0.38 to 2.38  0.37 to 5.47  0.97 to 1.06  0.59 to 2.15  0.98 to 1.07  0.97 to 1.08  1.00 to 1.07  1.00 to 1.20 | 1.00  1.01  1.06  1.73  2.69  1.02  1.27  1.00  1.06  1.03  1.15 | 1.00 to 1.00  0.90 to 1.14  1.01 to 1.11  1.07 to 2.80  0.97 to 7.44  0.99 to 1.06  0.77 to 2.08  0.96 to 1.04  1.02 to 1.10  1.00 to 1.06  1.07 to 1.24 |
| Arterial blood gases (average on day 1-3)  Arterial pH (per 0.1 points decrease)  Arterial pH on day 1 (per 0.1 points decrease)  Serum HCO_3_ (per 1 point decrease)  Serum HCO_3_ on day 1 (per 1 point decrease)  PaCO_2_, mm Hg^a^  PaO_2_, mm Hg^a^ | 1.14  1.12  1.10  1.07  0.97  1.00 | 0.77 to 1.69  0.84 to 1.50  1.03 to 1.17  1.02 to 1.13  0.94 to 1.00  0.99 to 1.01 | 1.60  1.23  1.13  1.11  0.98  1.00 | 1.13 to 2.26  0.93 to 1.63  1.07 to 1.20  1.05 to 1.16  0.96 to 1.01  0.99 to 1.01 | 1.90  1.41  1.16  1.11  0.98  1.00 | 1.55 to 2.33  1.22 to 1.61  1.11 to 1.21  1.07 to 1.14  0.96 to 1.00  0.99 to 1.01 |
| PaO_2_:FiO_2_ ratio^a^ | 1.00 | 1.00 to 1.01 | 1.00 | 1.00 to 1.00 | 1.00 | 1.00 to 1.00 |
| Oxygenation index^a^ | 1.00 | 0.97 to 1.04 | 1.02 | 0.99 to 1.04 | 1.02 | 1.01 to 1.03 |
| Fluid overload, median (IQR), %  Day 1  Day 2  Day 3 | 1.04  1.06  1.05 | 0.93 to 1.16  0.99 to 1.13  1.00 to 1.09 | 1.15  1.11  1.07 | 1.05 to 1.27  1.05 to 1.17  1.03 to 1.11 | 1.08  1.08  1.06 | 0.99 to 1.17  1.04 to 1.13  1.03 to 1.09 |

ACEI = angiotensin converting enzyme inhibitors, APACHE = Acute Physiology, Age, Chronic Health Evaluation, BMI = body mass index, CI = confidence interval, ECMO = extracorporeal membrane oxygenation, FiO_2_ = fractional of inspired oxygen, HFOV = high frequency oscillatory ventilation, NMBA = neuromuscular blocking agents, NSAIDs = non-steroidal anti-inflammatory drugs, PaCO_2_ = partial pressure of carbon dioxide in arterial blood, PaO_2_ = partial pressure of oxygen in arterial blood, PBW = predicted body weight, PEEP = positive end-expiratory pressure, RVSP = right ventricular systolic pressure, SHR = sub-distribution hazard ratio, SOFA = sequential organ failure assessment.

^a^per 1 point increase

**Table S5. Baseline Characteristics by Acute Kidney Injury in Acute Respiratory Distress Syndrome Patients without Septic Shock**

| Characteristics | No AKI (76) | | AKI (102) | | *p* | |
| --- | --- | --- | --- | --- | --- | --- |
| Age, mean (SD), years | 49.6 | (16.7) | 55.3 | (14) |  | 0.01^a^ |
| Male sex, n (%) | 37 | (48.7) | 59 | (57.8) |  | 0.23 |
| BMI, median (IQR), kg/m^2^ | 30.6 | (25.8- 36.2) | 32.5 | (26.9-41.2) |  | 0.21 |
| Race, n (%)  White  Black or African American | 62  11 | (81.6)  (14.5) | 70  32 | (68.6)  (31.4) |  | 0.05  0.01^a^ |
| SOFA, mean (SD), points | 8.3 | (2.4) | 9.9 | (2.9) | < | 0.001^a^ |
| Non-renal SOFA, mean (SD), points | 8.1 | (2.3) | 9.0 | (2.5) |  | 0.02^a^ |
| APACHE III, mean (SD), points | 89.5 | (23.5) | 99.5 | (27.2) |  | 0.01^a^ |
| Charlson comorbidities index, median (IQR), points | 2 | (0-4) | 3 | (1 -5) |  | 0.004^a^ |
| Comorbidities, n (%)  Chronic lung diseases  Diabetes  Active malignancies  Liver disease  Heart failure  Recent surgery within 3 months. | 28  15  9  3  4  3 | (36.8)  (19.7)  (11.8)  (3.9)  (5.3)  (3.9) | 37  36  18  5  13  4 | (36.3)  (35.3)  (17.6)  (4.9)  (12.7)  (3.9) |  | 0.94  0.02^a^  0.29  1.00  0.09  1.00 |
| Cause of ARDS, n (%)  Pneumonia  Aspiration  Non-pulmonary sepsis  Pancreatitis | 57  17  3  5 | (75)  (22.4)  (3.9)  (6.6) | 85  19  6  3 | (83.3)  (18.6)  (5.9)  (2.9) |  | 0.17  0.54  0.73  0.29 |
| Echocardiographic findings  EF, median (IQR), %  RVSP, median (IQR), mm Hg | 60  39 | (55-65)  (31-48) | 59  41 | (55-63.3)  (33.1-51.5) |  | 0.14  0.21 |
| Nephrotoxic agents, n (%)  Antimicrobial agents  Contrast agents  ACEI  Calcineurin inhibitors  NSAIDs | 67  25  13  5  4 | (88.2)  (32.9)  (17.1)  (6.6)  (5.3) | 79  26  15  4  2 | (82.3)  (25.7)  (14.7)  (3.9)  (2) |  | 0.29  0.30  0.66  0.50  0.40 |
| Number of nephrotoxic agents | 2 | (1-2) | 2 | (1-3) |  | 0.66 |
| Rescue therapies (%)  Continuous NMBA  Inhaled vasodilators  Prone positioning  Recruitment maneuvers  HFOV | 18  7  8  2  1 | (23.7)  (9.2)  (10.5)  (2.6)  (1.3) | 34  23  13  9  2 | (33.3)  (22.5)  (12.7)  (8.8)  (2) |  | 0.16  0.02^a^  0.65  0.12  1.00 |
| Known baseline SCr, n (%) | 33 | (43.4) | 57 | (55.9) |  | 0.10 |
| Baseline SCr, mean (SD), mg/dL | 0.82 | (0.19) | 0.86 | (0.18) |  | 0.26 |
| eGFR, median (IQR), mL/min per 1.73 m^2^ | 93.2 | (78.2-108.1) | 93.7 | (78.6-110.4) |  | 0.45 |
| Time from ARDS diagnosis to hospital admission (IQR), days | 0 | (-3 to 1) | 0 | (-2 to 2) |  | 0.18 |

ACEI = angiotensin converting enzyme inhibitors, AKI = acute kidney injury, APACHE = acute physiology, age, chronic health evaluation, ARDS = acute respiratory distress syndrome, BMI = body mass index, EF = ejection fraction, eGFR = estimated glomerular filtration rate, HFOV = high frequency oscillatory ventilation, IQR = interquartile range, NMBA = neuromuscular blocking agents, NSAIDs = non-steroidal anti-inflammatory drugs, RVSP = right ventricular systolic pressure, SCr = serum creatinine, SD = standard deviation, SOFA = sequential organ failure assessment.

^a^p < 0.05

**Table S6.** **Ventilator Settings Averaged on Day 1 to 3, Arterial Blood Gas, and Fluid Overload in Acute Respiratory Distress Syndrome Patients without Septic Shock by Acute Kidney Injury**

| Ventilator settings | No AKI (76) | | AKI (102) | | *p* | |
| --- | --- | --- | --- | --- | --- | --- |
| VT, median (IQR), mL | 456 | (411-512) | 489 | (433-546) |  | 0.04^a^ |
| VT, median (IQR), (mL/kg PBW) | 7.6 | (7.0-8.7) | 7.5 | (6.9- 8.5) |  | 0.80 |
| PEEP, median (IQR), cm H_2_O | 10 | (8-12) | 10 | (8-13.3) |  | 0.33 |
| FiO_2_, median (IQR) | 0.65 | (0.50-0.75) | 0.70 | (0.57-0.87) |  | 0.07 |
| Pplat, median (IQR), cm H_2_O | 25 | (22-30) | 28.5 | (23.8-34.6) |  | 0.04^a^ |
| Pplat > 30 cm H_2_O (%) | 9 | (23.1) | 25 | (44.6) |  | 0.03^a^ |
| DP, median (IQR), cm H_2_O | 15 | (11.8-18.5) | 15 | (12.4-21.1) |  | 0.59 |
| Mean airway pressure, median (IQR),  cm H_2_O | 16.7 | (13-18.3) | 17.5 | (14.6-21.4) |  | 0.05 |
| Ppeak, mean (SD), cm H_2_O | 29.1 | (7) | 31.2 | (6.5) |  | 0.08 |
| Minute ventilation, median (IQR), L/min | 10.3 | (8.4-11.9) | 10.5 | (9.1-12.4) |  | 0.24 |
| Lung protective ventilation, n (%)^b^ | 18 | (36.7) | 24 | (32.4) |  | 0.62 |
| Arterial blood gases |  |  |  |  |  |  |
| Arterial pH, median (IQR) | 7.39 | (7.35-7.43) | 7.37 | (7.31-7.42) |  | 0.01^a^ |
| Arterial pH on day 1 | 7.37 | (7.33-7.41) | 7.35 | (7.3-7.4) |  | 0.10 |
| Serum HCO_3_ median (IQR), mEq/L | 25 | (22.5-28.5) | 24 | (20.7- 27) |  | 0.04^a^ |
| Serum HCO_3_ on day 1 | 26 | (22-29) | 23.5 | (21-28) |  | 0.07 |
| Serum lactate, median (IQR), mmol/L | 1.6 | (1.1-2.0) | 1.5 | (1.1-1.9) |  | 0.81 |
| Serum lactate on day 1 | 1.7 | (1.3-2.4) | 1.5 | (1.1-2.0) |  | 0.31 |
| PaCO_2_, median (IQR), mm Hg | 45 | (38.6-49.4) | 44 | (38.4-50.8) |  | 0.90 |
| PaO_2_, median (IQR), mm Hg | 87.6 | (77.3-104) | 85.2 | (74.2-112) |  | 0.68 |
| PaO_2_:FiO_2_, median (IQR) | 144 | (109-185) | 135 | (98-177) |  | 0.13 |
| Oxygenation index, median (IQR) | 11.2 | (6.7-16) | 14.5 | (9.7-23.9) |  | 0.01 |
| Fluid overload, median (IQR), %  Day 1  Day 2  Day 3 | 0.1  1.2  1.3 | (-0.8 to 2.6)  (-0.2 to 2.9)  (-0.5 to 4.9) | 0.3  1.9  3.2 | (-0.6 to 2.6)  (-0.1 to 5.6)  (0.9 to 6.8) |  | 0.51  0.25  0.04 |

AKI = acute kidney injury, DP = driving pressure, FiO_2_ = fraction of inspired oxygen, HCO_3_ = bicarbonate, IQR = interquartile range, PaCO_2_ = partial pressure of carbon dioxide in arterial blood, PaO_2_ = partial pressure of oxygen in arterial blood, PBW = predicted body weight, PEEP = positive end-expiratory pressure, Ppeak = peak inspiratory pressure, Pplat = plateau pressure, SD = standard deviation, VT = tidal volume.

^a^p < 0.05

^b^Tidal volume ≤ 8 mL/kg PBW and inspiratory plateau pressure ≤ 30 cm H_2_O

**Table S7. Baseline Characteristics by Severity of Acute Kidney Injury in Acute Respiratory Distress Syndrome Patients without Septic Shock**

| Characteristics | No AKI  (76) | | Stage I AKI  (33) | | Stage II AKI  (35) | | Stage III AKI  (34) | | *p* | |
| --- | --- | --- | --- | --- | --- | --- | --- | --- | --- | --- |
| Age, mean (SD), years | 49.6 | (16.7) | 56.4 | (15.8) | 55 | (14.5) | 54.4 | (11.8) |  | 0.14 |
| Male sex, n (%) | 37 | (48.7) | 19 | (57.6) | 22 | (62.9) | 18 | (52.9) |  | 0.54 |
| BMI, median (IQR), kg/m^2^ | 30.6 | (25.8- 36.2) | 31.5 | (26.9-37.1) | 30.7 | (25.5- 42.9) | 35.2 | (29.8-42.2) |  | 0.15 |
| Race, n (%)  White  Black or African American | 62  11 | (81.6)  (14.5) | 21  12 | (63.6)  (36.4) | 23  12 | (65.7)  (34.3) | 26  8 | (76.5)  (23.5) |  | 0.14  0.04 |
| SOFA, mean (SD), points | 8.3 | (2.4) | 9.1 | (2.7) | 9.9 | (3)^a^ | 10.7 | (3)^a^ | < | 0.001 |
| Non-renal SOFA, mean (SD), points | 8.1 | (2.3) | 8.5 | (2.6) | 9.1 | (2.5) | 9.3 | (2.4) |  | 0.05 |
| APACHE III, mean (SD), points | 89.5 | (23.5) | 88 | (23.3) | 101.2 | (22.8) | 108.9 | (31.3)^a, b^ | < | 0.001 |
| Charlson comorbidities index, median (IQR), points | 2 | (0-4) | 2 | (1-4 ) | 3 | (1-4) | 3 | (1-5)^a^ |  | 0.02 |
| Comorbidities, n (%)  Chronic lung diseases  Diabetes  Active malignancies  Liver disease  Heart failure  Recent surgery within 3 months. | 28  15  9  3  4  3 | (36.8)  (19.7)  (11.8)  (3.9)  (5.3)  (3.9) | 10  10  3  0  4  1 | (30.3)  (30.3)  (9.1)  (0)  (12.1)  (3) | 14  12  5  2  7  2 | (40)  (34.3)  (14.3)  (5.7)  (20)  (5.7) | 13  14  10  3  2  1 | (38.2)  (41.2)  (29.4)  (8.8)  (5.9)  (2.9) |  | 0.85  0.10  0.07  0.32  0.09  0.95 |
| Cause of ARDS, n (%)  Pneumonia  Aspiration  Non-pulmonary sepsis  Pancreatitis | 57  17  3  5 | (75)  (22.4)  (3.9)  (6.6) | 28  8  4  1 | (84.8)  (24.2)  (12.1)  (3) | 30  7  1  1 | (85.7)  (20)  (2.9)  (2.9) | 27  4  1  1 | (79.4)  (11.8)  (2.9)  (2.9) |  | 0.50  0.56  0.27  0.78 |
| Echocardiographic findings  EF, median (IQR), %  RVSP, median (IQR), mm Hg | 60  39 | (55-65)  (31-48) | 55  37.5 | (55-60)  (28.5-45.5) | 60  42.5 | (55-63)  (37.8-51.5) | 60  41 | (55-66.7)  (34.6-54) |  | 0.13  0.19 |
| Nephrotoxic agents, n (%)  Antimicrobial agents  Contrast agents  ACEI  Calcineurin inhibitors  NSAIDs | 67  25  13  5  4 | (88.2)  (32.9)  (17.1)  (6.6)  (5.3) | 30  10  7  0  0 | (90.9)  (30.3)  (21.2)  (0)  (0) | 28  7  6  3  2 | (82.4)  (20)  (17.1)  (8.6)  (5.7) | 21  9  2  1  0 | (72.4)  (27.3)  (5.9)  (2.9)  (0) |  | 0.17  0.57  0.33  0.40  0.36 |
| Number of nephrotoxic agents | 2 | (1-2) | 2 | (1-3) | 2 | (1-3) | 2 | (0-2) |  | 0.66 |
| Rescue therapies (%)  Continuous NMBA  Inhaled vasodilators  Prone positioning  Recruitment maneuvers  HFOV | 18  7  8  2  1 | (23.7)  (9.2)  (10.5)  (2.6)  (1.3) | 6  3  2  2  1 | (18.2)  (9.1)  (6.1)  (6.1)  (3) | 12  8  4  2  1 | (34.3)  (22.9)  (11.4)  (5.7)  (2.9) | 16  12  7  5  0 | (47.1)  (35.3)^a^  (20.6)  (14.7)  (0) |  | 0.03  0.003  0.34  0.11  0.59 |
| Known baseline SCr, n (%) | 33 | (43.4) | 18 | (54.5) | 23 | (65.7) | 16 | (47.1) |  | 0.16 |
| Baseline SCr, mean (SD), mg/dL | 0.82 | (0.19) | 0.83 | (0.18) | 0.88 | (0.22) | 0.85 | (0.14) |  | 0.66 |
| eGFR, median (IQR), mL/min per 1.73 m^2^ | 93.2 | (78.2-108.1) | 87.6 | (76.1-114.5) | 97.5 | (81.8-107.7) | 92.7 | (79.6-110) |  | 0.90 |
| Time from ARDS diagnosis to hospital admission (IQR), days | 0 | (-3 to 1) | 0 | (-1 to 3) | 0 | (-1 to 1.5) | 0 | (-3 to 1.8) |  | 0.16 |

ACEI = angiotensin converting enzyme inhibitors, AKI = acute kidney injury, APACHE = acute physiology, age, chronic health evaluation, ARDS = acute respiratory distress syndrome, BMI = body mass index, EF = ejection fraction, eGFR = estimated glomerular filtration rate, HFOV = high frequency oscillatory ventilation, IQR = interquartile range, NMBA = neuromuscular blocking agents, NSAIDs = non-steroidal anti-inflammatory drugs, RVSP = right ventricular systolic pressure, SCr = serum creatinine, SD = standard deviation, SOFA = sequential organ failure assessment.

^a^p < 0.05 when compared with patients without AKI.

^b^p < 0.05 when compared with patients with stage I AKI.

**Table S8.** **Ventilator Settings Averaged on Day 1 to 3, Arterial Blood Gas, and Fluid Overload in Acute Respiratory Distress Syndrome Patients without Septic Shock by Severity of Acute Kidney Injury**

| Ventilator settings | No AKI  (76) | | Stage I AKI  (33) | | Stage II AKI  (35) | | Stage III AKI  (34) | | *p* | |
| --- | --- | --- | --- | --- | --- | --- | --- | --- | --- | --- |
| VT, median (IQR), mL | 456 | (411-512) | 488 | (414-559) | 499 | (432-544) | 487 | (443-544) |  | 0.25 |
| VT, median (IQR), (mL/kg PBW) | 7.6 | (7.0-8.7) | 7.5 | (6.8-8.0) | 7.3 | (7.0-8.1) | 7.9 | (7.0-9.2) |  | 0.42 |
| PEEP, median (IQR), cm H_2_O | 10 | (8-12) | 10 | (7-13.4) | 9.8 | (8.4-11.9) | 12 | (8-14) |  | 0.64 |
| FiO_2_, median (IQR) | 0.65 | (0.50-0.75) | 0.7 | (0.57-0.82) | 0.69 | (0.50-0.85) | 0.73 | (0.59-0.89) |  | 0.23 |
| Pplat, median (IQR), cm H_2_O | 25 | (22-30) | 28 | (22.7-34) | 29 | (24.3-33.5) | 29.3 | (24.1-36.3) |  | 0.23 |
| Pplat > 30 cm H_2_O (%) | 9 | (23.1) | 8 | (47.1) | 9 | (39.1) | 8 | (50) |  | 0.16 |
| DP, median (IQR), cm H_2_O | 15 | (11.8-18.5) | 16 | (12-19) | 16 | (12.8- 21.8) | 13 | (12.5-18) |  | 0.85 |
| Mean airway pressure, median (IQR), cm H_2_O | 16.7 | (13-18.3) | 17.3 | (14-20.7) | 15.7 | (14.7-22) | 19 | (14.8-21.9) |  | 0.25 |
| Ppeak, mean (SD), cm H_2_O | 29.1 | (7) | 29.6 | (4.8) | 31.9 | (7) | 31.9 | (7.5) |  | 0.15 |
| Minute ventilation, median (IQR), L/min | 10.3 | (8.4-11.9) | 10.4 | (9.2-11.8) | 10.5 | (8.7-12.8) | 11.4 | (9.4- 12.7) |  | 0.61 |
| Lung protective ventilation, n (%)^c^ | 18 | (36.7) | 7 | (31.8) | 11 | (39.3) | 6 | (25) |  | 0.70 |
| Arterial blood gas |  |  |  |  |  |  |  |  |  |  |
| Arterial pH, median (IQR) | 7.39 | (7.35-7.43) | 7.39 | (7.35- 7.44) | 7.35 | (7.3-7.41) | 7.36 | (7.29-7.38)^a, b^ |  | 0.003 |
| Arterial pH on day 1 | 7.37 | (7.33-7.41) | 7.37 | (7.32-7.44) | 7.35 | (7.29-7.37) | 7.34 | (7.27-7.38) |  | 0.03 |
| Serum HCO_3_, median (IQR), mEq/L | 25 | (22.5-28.5) | 24.8 | (22-27.7) | 23.4 | (21-25.5) | 23.5 | (19.8-27) |  | 0.08 |
| Serum HCO_3_ on day 1 | 26 | (22-29) | 25 | (21-28) | 23 | (20-26.3) | 22 | (20.5-27) |  | 0.22 |
| Serum lactate, median (IQR), mmol/L | 1.6 | (1.1-2.0) | 1.5 | (1.2-1.8) | 1.6 | (1.2-2.1) | 1.4 | (1.1-1.8) |  | 0.77 |
| Serum lactate on day 1 | 1.7 | (1.3-2.4) | 1.6 | (1.3-1.9) | 1.5 | (1.2-1.9) | 1.5 | (1.1-2.8) |  | 0.78 |
| PaCO_2_, median (IQR), mm Hg | 45 | (38.6-49.4) | 42.5 | (39.3-47.8) | 45.7 | (38.8-52.6) | 46.3 | (38.3- 51.3) |  | 0.76 |
| PaO_2_, median (IQR), mm Hg | 87.6 | (77.3-104) | 89.1 | (75.3-112.1) | 84 | (71.2-94.8) | 84.3 | (72.8-123.8) |  | 0.69 |
| PaO_2_:FiO_2_, median (IQR) | 144 | (109-185) | 139 | (113- 185) | 134 | (97-163) | 134 | (91-181) |  | 0.29 |
| Oxygenation index, median (IQR) | 11.2 | (6.7-16) | 15.6 | (9.8-19.3) | 13 | (9.7-23.9) | 16.2 | (9.2-25.5) |  | 0.09 |
| Fluid overload, median (IQR), %  Day 1  Day 2  Day 3 | 0.1  1.2  1.3 | (-0.8 to 2.6)  (-0.2 to 2.9)  (-0.5 to 4.9) | -0.1  0.7  3.2 | (-0.8 to 2.9)  (-0.5 to 4)  (-0.1 to 4.3) | 0.2  2.4  3.5 | (-0.5 to 2.5)  (-0.2 to 5.4)  (0.2 to 7.4) | 1.45  3  3.4 | (-0.2 to 2.6)  (0.5 to 5.9)  (1.9 to 7.0) |  | 0.63  0.33  0.13 |

AKI = acute kidney injury, DP = driving pressure, FiO_2_ = fraction of inspired oxygen, HCO_3_ = bicarbonate, IQR = interquartile range, PaCO_2_ = partial pressure of carbon dioxide in arterial blood, PaO_2_ = partial pressure of oxygen in arterial blood, PBW = predicted body weight, PEEP = positive end-expiratory pressure, Ppeak = peak inspiratory pressure, Pplat = plateau pressure, SD = standard deviation, VT = tidal volume.

^a^p < 0.05 when compared with patients without AKI.

^b^p < 0.05 when compared with patients with stage I AKI.

^c^Tidal volume ≤ 8 mL/kg PBW and inspiratory plateau pressure ≤ 30 cm H_2_O

**Table S9. Multivariable Competing Risk Regression for Factors Associated with the Development of Acute Kidney Injury in ARDS Patients without Septic Shock**

| Variable | SHR^b^ | 95% confidence interval | p | |
| --- | --- | --- | --- | --- |
| Age (year)^a^ | 1.02 | 1.00 to 1.03 |  | 0.01 |
| SOFA score^a^ | 1.18 | 1.10 to 1.27 | < | 0.001 |
| History of diabetes mellitus | 1.59 | 1.05 to 2.41 |  | 0.03 |

SHR = sub-distribution hazard ratio, SOFA = sequential organ failure assessment.

^a^per 1 point increase

^b^Pool analysis after multivariable competing risk regression with regression Fine and Gray model of 50 sets of multiple imputed data

**Table S10. Multivariable Competing Risk Regression for Factors Associated with the Development of Severity of Acute Kidney Injury in Acute Respiratory Distress Syndrome Patients without Septic Shock**

| Variable | SHR^b^ | 95% confidence interval | *p* | |
| --- | --- | --- | --- | --- |
| Stage I AKI |  |  |  | |
| Ejection fraction | 0.97 | 0.93 to 1.00 | 0.03 | |
| Stage II AKI |  |  |  | |
| SOFA score ^a^ | 1.22 | 1.09 to 1.38 |  | 0.001 |
| History of heart failure | 6.50 | 2.46 to 17.19 | < | 0.001 |
| Serum HCO_3_ on day 1 (per 1 unit decrease) | 1.11 | 1.03 to 1.19 |  | 0.01 |
| Stage III AKI |  |  |  |  |
| SOFA score ^a^ | 1.25 | 1.09 to 1.43 |  | 0.001 |
| Charlson comorbidity index ^a^ | 1.18 | 1.04 to 1.32 |  | 0.01 |
| Arterial pH on day 1 (per 0.1 units decrease) | 1.34 | 1.06 to 1.70 |  | 0.02 |
| Inhaled vasodilators | 2.52 | 1.18 to 5.39 |  | 0.02 |

SHR = sub-distribution hazard ratio, SOFA = sequential organ failure assessment.

^a^per 1 point increase

^b^Pool analysis after multivariable competing risk regression with regression Fine and Gray model of 50 sets of multiple imputed data

**Table S11. Sensitivity Analysis of Non-Imputed Data Using Multivariable Competing Risk Regression for Factors Associated with the Development of Acute Kidney Injury and Severity of Acute Kidney Injury in Acute Respiratory Distress Syndrome Patients**

| Variable | SHR | 95% confidence interval | *p* | |
| --- | --- | --- | --- | --- |
| Development of AKI  SOFA score^a^  Charlson comorbidity index^a^  Arterial pH on day 1 (per 0.1 unit decrease) | 1.16  1.08  1.23 | 1.11 to 1.21  1.02 to 1.14  1.09 to 1.38 | <  < | 0.001  0.01  0.001 |
| Stage I AKI  Age^a^  Serum HCO_3_ on day 1 (per 1 unit decrease) | 1.03  1.08 | - 1. to 1.05   2. to 1.13 | < | 0.001  0.002 |
| Stage II AKI  Age^a^  Serum HCO_3_ on day 1 (per 1 unit decrease)  SOFA score^a^  History of heart failure  Average peak airway pressure on day 1-3^a^ | 1.03  1.12  1.21  6.36  1.04 | - 1. to 1.04   1.06 to 1.18  1.11 to 1.33  2.69 to 15.03  1.00 to 1.08 | <  <  < | 0.01  0.001  0.001  0.001  0.03 |
| Stage III AKI  SOFA score^a^  Arterial pH on day 1 (per 0.1 unit decrease)  Body mass index^a^  Charlson comorbidity index^a^ | 1.31  1.41  1.04  1.14 | 1.23 to 1.39  1.21 to 1.63  1.02 to 1.05  1.05 to 1.24 | <  <  < | 0.001  0.001  0.001  0.002 |

AKI = acute kidney injury, HCO3 = bicarbonate, SHR = sub-distribution hazard ratio, SOFA = sequential organ failure assessment. ^a^per 1 point increase

**
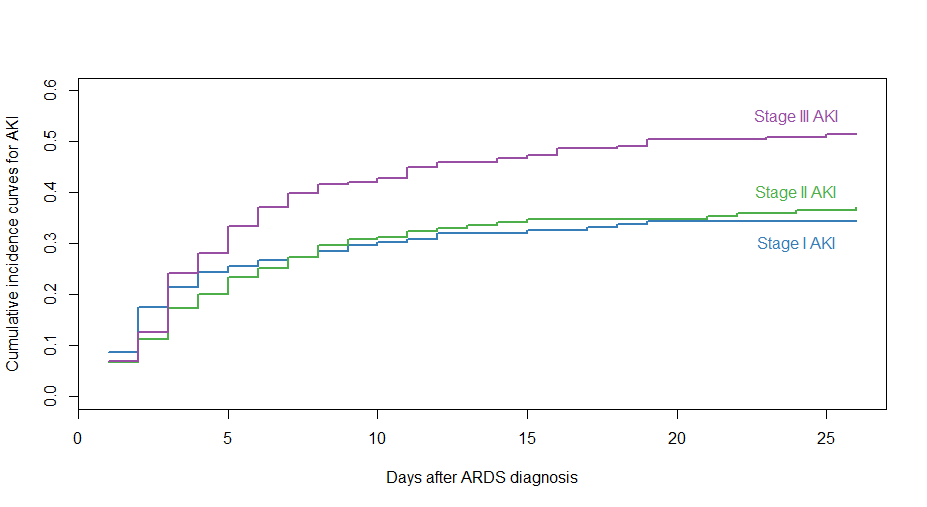
**

**Figure S1. Cumulative incidence curves for development acute kidney injury (AKI) by AKI severities.**

**References**

1. White IR, Royston P, Wood AM. Multiple imputation using chained equations: Issues and guidance for practice. Stat Med. 2011;30(4):377-99.

2. Moons KG, Donders RA, Stijnen T, Harrell FE, Jr. Using the outcome for imputation of missing predictor values was preferred. J Clin Epidemiol. 2006;59(10):1092-101.

3. van Buuren S, Groothuis-Oudshoorn K. mice: Multivariate Imputation by Chained Equations in R. J Stat Softw. 2011;45(3):1-67.
